# Synthesis, Characterization, and Potential Usefulness in Liver Function Assessment of Novel Bile Acid Derivatives with Near-Infrared Fluorescence (NIRBAD)

**DOI:** 10.1021/acs.bioconjchem.4c00168

**Published:** 2024-07-03

**Authors:** Alvaro G. Temprano, Beatriz Sanchez de Blas, Concepción Pérez-Melero, Ricardo Espinosa-Escudero, Oscar Briz, Paula Cinca-Fernando, Lucia Llera, Maria J. Monte, Francisco A. Bermejo-Gonzalez, Jose J.G. Marin, Marta R. Romero

**Affiliations:** † Experimental Hepatology and Drug Targeting (HEVEPHARM), University of Salamanca, IBSAL, Salamanca 37007, Spain; ‡ Center for the Study of Liver and Gastrointestinal Diseases (CIBEREHD), Carlos III National Institute of Health, Madrid 28029, Spain; § Pharmaceutical Chemistry Laboratory, Pharmaceutical Sciences Department, University of Salamanca, IBSAL, Salamanca 37007, Spain; ∥ Organic Chemistry, School of Chemistry, University of Salamanca, Salamanca 37007, Spain

## Abstract

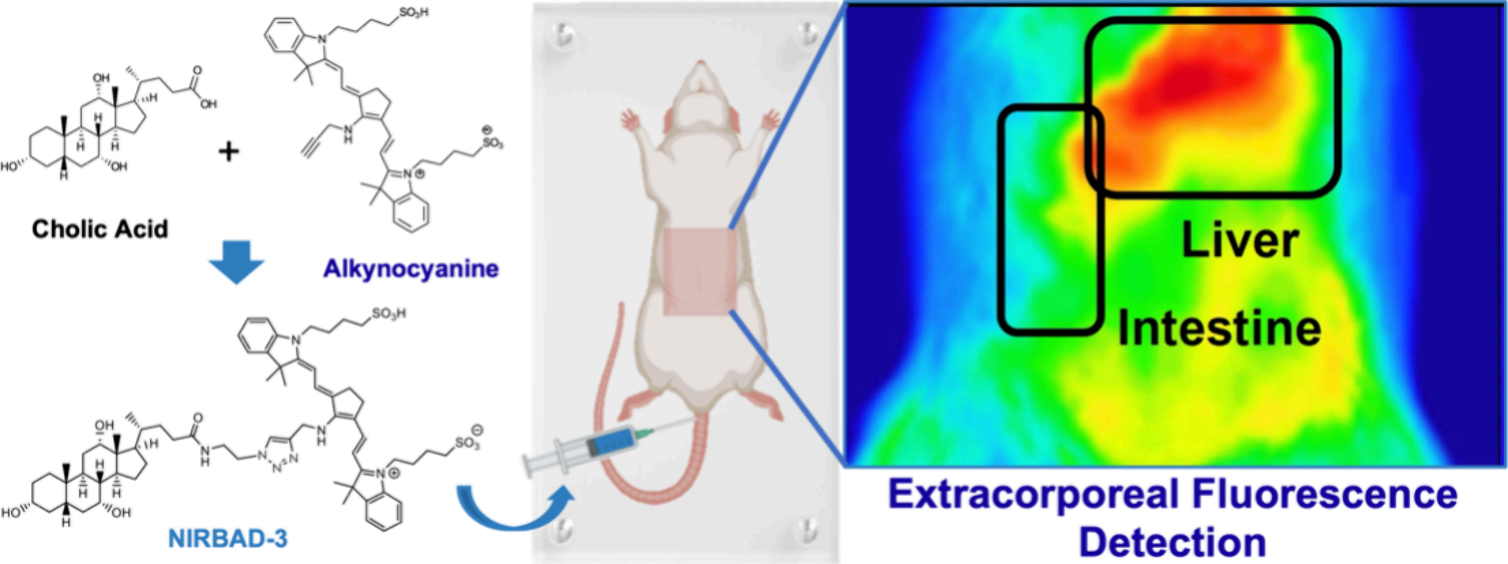

Conventional serum markers often fail to accurately detect cholestasis accompanying many liver diseases. Although elevation in serum bile acid (BA) levels sensitively reflects impaired hepatobiliary function, other factors altering BA pool size and enterohepatic circulation can affect these levels. To develop fluorescent probes for extracorporeal noninvasive hepatobiliary function assessment by real-time monitoring methods, 1,3-dipolar cycloaddition reactions were used to conjugate near-infrared (NIR) fluorochromes with azide-functionalized BA derivatives (BAD). The resulting compounds (NIRBADs) were chromatographically (FC and PTLC) purified (>95%) and characterized by fluorimetry, ^1^H NMR, and HRMS using ESI ionization coupled to quadrupole TOF mass analysis. Transport studies using CHO cells stably expressing the BA carrier NTCP were performed by flow cytometry. Extracorporeal fluorescence was detected in anesthetized rats by high-resolution imaging analysis. Three NIRBADs were synthesized by conjugating alkynocyanine 718 with cholic acid (CA) at the COOH group via an ester (NIRBAD-1) or amide (NIRBAD-3) spacer, or at the 3α-position by a triazole link (NIRBAD-2). NIRBADs were efficiently taken up by cells expressing NTCP, which was inhibited by taurocholic acid (TCA). Following i.v. administration of NIRBAD-3 to rats, liver uptake and consequent release of NIR fluorescence could be extracorporeally monitored. This transient organ-specific handling contrasted with the absence of release to the intestine of alkynocyanine 718 and the lack of hepatotropism observed with other probes, such as indocyanine green. NIRBAD-3 administration did not alter serum biomarkers of hepatic and renal toxicity. NIRBADs can serve as probes to evaluate hepatobiliary function by noninvasive extracorporeal methods.

## Highlights

1. Novel near-infrared bile acid derivatives (NIRBADs) were synthesized using click chemistry.
2. NIRBADs are taken up by the bile acid transporter NTCP, which is inhibited by taurocholic acid.
3. After i.v. administration to rats at nontoxic doses, NIRBAD-3 was rapidly taken up by the liver and secreted into bile.
4. Hepatic handling of NIRBAD-3 can be monitored by extracorporeal detection of NIR fluorescence.

## Introduction

Bile acids (BAs) are steroids synthesized by the liver from cholesterol. These compounds are characterized by their marked organotropism toward tissues of the so-called enterohepatic circuit. BA vectorial properties are due to the presence in hepatocytes and intestinal epithelial cells (mainly in the ileum) of transmembrane proteins accounting for highly effective BA uptake from sinusoidal blood and the intestinal lumen, respectively. Although Na^+^-independent transporters, belonging to the OATP family, are involved in BA uptake by hepatocytes, this process mainly occurs via Na^+^-dependent transporters encoded by a member of the family 10 of the solute carrier (SLC) superfamily of genes, namely, the hepatic Na^+^-taurocholate cotransporting polypeptide (NTCP, *SLC10A1*). The intestinal apical sodium-dependent BA transporter (ASBT, *SLC10A2*) plays a similar role in the ileum.^1^ Owing to the high affinity and specificity of both transporters for BA as substrates, these compounds have been used as shuttles for molecules with pharmacological properties targeted toward tissues within this circuit, such as chlorambucil,^2^ nucleosides,^3^ nitrogenous bases,^4^ polyamines,^5^ or cisplatin,^6,7^ thus increasing their bioavailability in the liver and intestine while reducing adverse side effects.^8^

Moreover, in the assessment of liver function within clinical settings, diverse methodologies leveraging labeled BA derivatives (BADs) have been explored (for a recent review, see ref (9)). These include ^18^F-labeled BA derivatives as positron emitter tomography (PET) tracers to study hepatic transporters.^10^ It is noteworthy that currently used approaches for the diagnosis of several hepatobiliary disorders (e.g., focal lesions, tumors, and cholestasis) also include cholescintigraphy using ^99m^Tc-labeled iminodiacetic acid derivatives and magnetic resonance imaging (MRI) using hepatocyte-specific contrast agents, such as gadoxetate. However, all probes mentioned lack enterohepatic vectoriality and consequently lack tissue specificity.^11^

To overcome this limitation, a wide variety of fluorescent BADs have been synthesized. This includes several amido fluorescein (amF) derivatives, such as cholyl-amF, cholyl-glycyl-amF (CGamF), chenodeoxycholyl-glycyl-amF, and ursodeoxycholyl-glycyl-amF, which have been used with *in vitro* and *in vivo* models to study BA transport, drug–BA interactions, and cytosol-nucleus traffic.^12,13^ They have facilitated the exploration of BA organotropism, and the usefulness of drug targeting of BAD vectorized toward cells expressing BA transporters.^6,14^ Besides, cholyl-l-lysyl-fluorescein (CLF) has been used *in vivo* in combination with intravital imaging performed through confocal microscopy in anesthetized animals for assessing liver function.^15^ Dansylated cholic acid (CA) derivatives have proven valuable for determining BA amphipathic characteristics and aggregation behavior, as well as their binding to proteins and their handling by the liver.^16^

All the fluorescent BADs mentioned above emit in the visible range of the spectrum, which limits their usefulness. The present study aimed to synthesize novel compounds (NIRBADs) labeled with tags emitting near-infrared fluorescence (NIR, 780–2500 nm) with greater tissue penetration and enhanced liver organotropism due to their conjugation with BADs. The aim of synthesizing these novel compounds was to enhance their usefulness by adding the advantage of enabling their visualization from outside the body without the need for invasive procedures.

## Results and Discussion

Molecules with NIR fluorescence are helpful tools in clinical practice. For instance, indocyanine green (ICG) has been used to carry out extracorporeal detection of lymph nodes and vessels in cancer and other diseases with an ischemic profile, to study the liver function in patients with hepatic tumors before surgical removal and during laparoscopic cholecystectomy for the identification of biliary anatomy.^17^ However, ICG, like other available NIR probes, has the limitation of lacking tissue-selective characteristics. In contrast, the NIR probes synthesized here are cholephilic compounds, i.e., efficiently taken up by hepatocytes and secreted into bile.

Triazoles have proven helpful in generating a broad range of molecules with biological activity.^18,19^ These compounds are synthesized by the copper-catalyzed Huisgen 1,3-dipolar cycloaddition, the most popular example of the click reaction, which involves alkynes and azides.^20^ Using 1,2,3-triazole moieties as linkers offers advantages due to their stability under typical physiological conditions and their ability to form hydrogen bonds. Additionally, the capacity of 1,2,3-triazoles to mimic the topological and electronic features of amide bonds makes them well suited for designing peptidomimetics with enhanced medicinal properties.^18,21^

**Figure 1:**
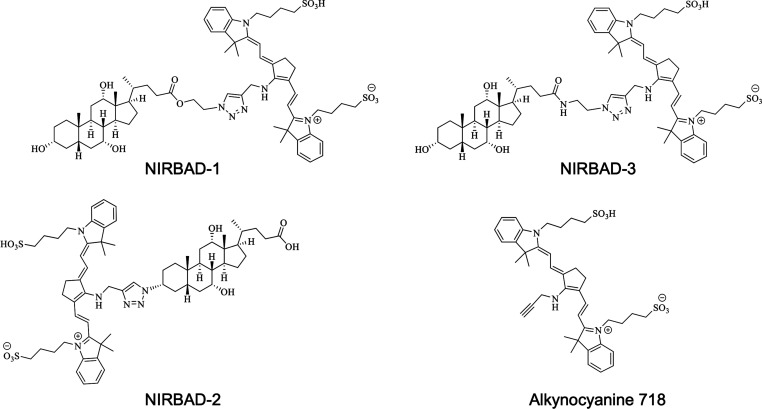
Structure of the new near-infrared bile acid derivatives (NIRBAD-1, NIRBAD-2, and NIRBAD-3) and the alkynocyanine 718 used as the fluorescent probe to synthesize NIRBADs.

Previous studies have established the click strategy’s efficacy in producing BA derivatives by binding them to active molecules with diverse residues.^18^ An example has been BA binding to peptides to obtain novel compounds in supramolecular chemistry.^22^ Additionally, significant strides have been made in the field of antimicrobial development through the binding of BA to beta-lactams, resulting in the synthesis of innovative antibiotic agents.^23^ Furthermore, clickable conjugates of BAs and nucleosides have been synthesized and assayed *in vitro* as anticancer and antituberculosis agents.^19^ In the present study, we leveraged this background to synthesize a new family of compounds (NIRBADs) (Figure 1) targeted to the enterohepatic circuit through a BA moiety and capable of emitting NIR fluorescence due to the linked fluorochrome, alkynocyanine 718. This compound was selected for this purpose due to two characteristics: (i) near-infrared emission wavelength (Ex664/Em718 nm) and (ii) a terminal triple bond, which eliminated the need for structural modifications prior to carrying out the envisioned 1,3-dipolar cycloaddition reaction with BA-azide derivatives.

**Figure 2:**
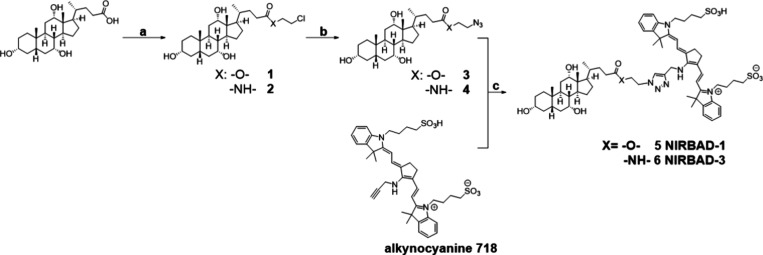
Reagents and conditions: (a) DCC, DMAP, for 1: (X = −O−), 2-chloroethanol, CH_3_Cl, 25 °C, 24 h, 95% and for 2 (R = −NH−), 2-chloroethanamine, CHCl_3_, 25 °C, 24 h, 95%; (b) NaN_3_, DMF, 100 °C, 48 h, 95% for **3** and 92% for **4**; (c) CuSO_4_, sodium ascorbate, ethanol, 25 °C, 7 days, 15% for **5** and 20% for **6**. Spectroscopic data for compounds **1** to **6** are shown in Figures S1 to S6.

NIRBAD-1 and NIRBAD-3 were obtained through a three-step reaction process (Figure 2). In the first step, CA reacts with 2-chloroethanol or 2-chloroethanamine to yield the corresponding compounds (**1**) or (**2**), respectively. Second, a S_N_2 substitution with sodium azide generates the key intermediate azide derivatives (**3**) and (**4**). Finally, a copper-catalyzed 1,3-dipolar cycloaddition reaction between azides 3/4 and alkynocyanine 718 provided the conjugate compounds (**5**) NIRBAD-1 and (**6**) NIRBAD-3.

**Figure 3:**
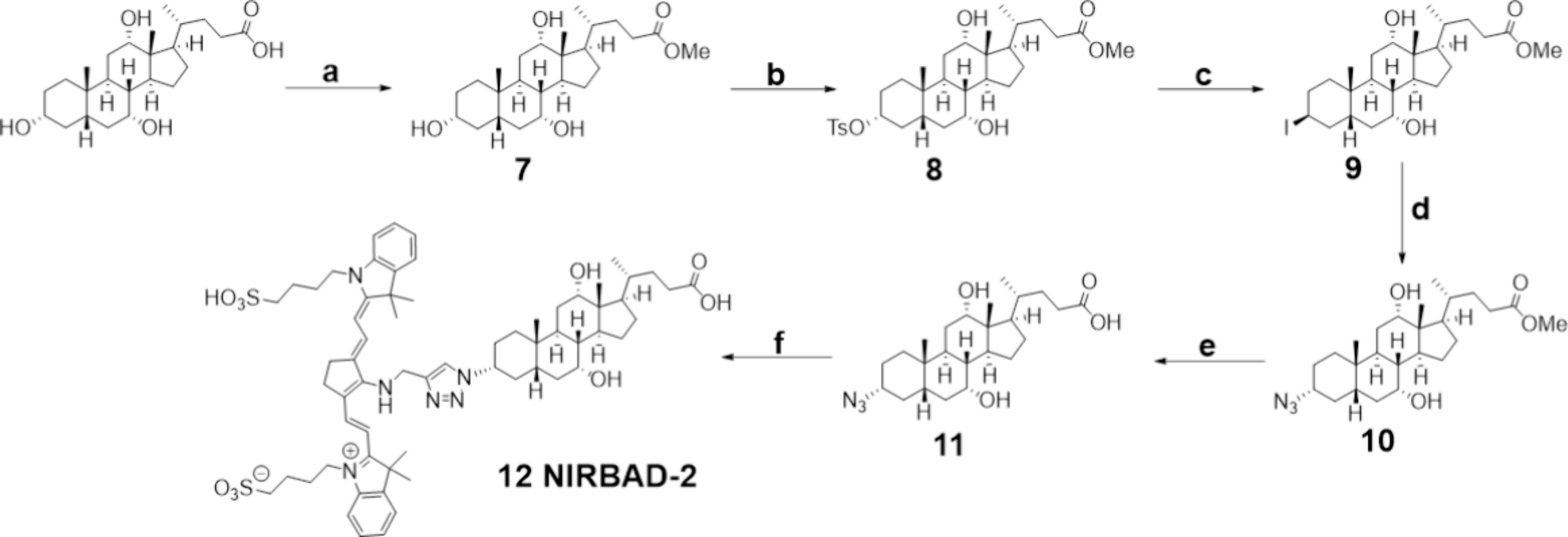
Reagents and conditions: (a) SOCl_2_, MeOH, 65 °C, 2 h, 70%; (b) TsCl, Pyr, 25 °C, 24 h, 37%; (c) KI, DMF, 92 °C, 1.5 h, 82%; (d) NaN_3_, DMF, 60 °C, 24 h, 77%; (e) LiOH, MeOH, 25 °C, 24 h, 71%; (f) CuSO_4_, alkynocyanine 718, sodium ascorbate, ethanol, 25 °C, 7 days, 20%. Spectroscopic data for compounds **7** to **12** are shown in Figures S7−S12.

For the synthesis of NIRBAD-2 (Figure 3), functionalization of the hydroxyl group at the 3α position of the CA steroid core was performed. First, the carboxylic acid of CA was protected by Fischer esterification using thionyl chloride in methanol, obtaining the corresponding methyl ester (**7**). Next, due to the basic character of the hydroxide anion making it a poor leaving group, the tosylation of the hydroxyl group at position **3** was carried out to obtain the tosyl derivative of CA (**8**). Then, an S_N_2 nucleophilic substitution reaction with inversion of the configuration of the tosylate group by iodine was performed, using KI and DMF as the solvent (**9**), resulting in the inversion of C3 configuration from *R* to *S*. Finally, to preserve the original hydroxyl configuration *R,* a subsequent S_N_2 substitution of the iodine was performed using sodium azide in DMF, resulting in the (3*R*) isomer (**10**) due to the new inversion of the configuration. The next step was the deprotection of the carboxylic acid by a basic hydrolysis reaction with LiOH in methanol (**11**). Finally, the 1,3-dipolar cycloaddition reaction between the azide derived (**11**) from CA and alkynocyanine 718 was performed. Once the crude reaction product was obtained, it was purified by preparative thin layer chromatography (PTLC) to obtain the purified NIRBAD-2 compound (**12**).

The synthesis of these compounds provides insight into their distinct properties arising from structural variations. Although NIRBAD-1 and NIRBAD-3 have undergone functionalization on their side chains, this has been achieved through chemically different linkers, i.e., an ester and an amide, respectively. Consequently, their biodistribution is anticipated to exhibit varying profiles due to the change of a hydrogen bond acceptor to a donor. Moreover, it is worth noting that esters and amides undergo metabolism at significantly different rates, further contributing to the potential dissimilarities.^24^ In contrast, NIRBAD-2 has been functionalized at the hydroxyl in the C3 position, maintaining the side chain in a carboxylate form. This distinction is also expected to influence its biodistribution. Furthermore, the stereochemistry of the chiral centers in the original BA, CA, has been preserved to retain its native structure. This characteristic ensures that the synthesized BA derivative is still efficiently recognized as a substrate by BA transporters.

**Figure 4:**
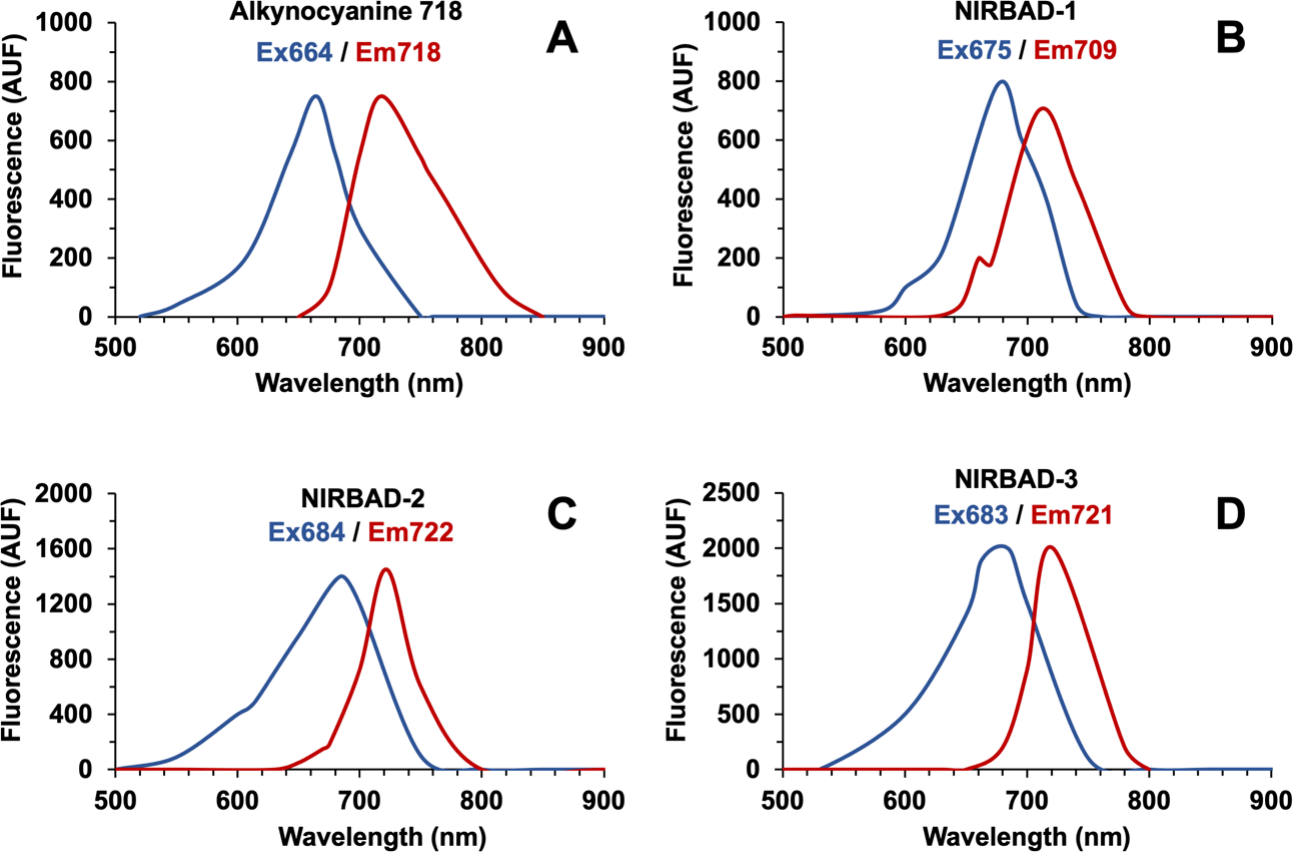
Excitation (blue) and emission (red) spectra of alkynocyanine 718 (A), NIRBAD-1 (B), NIRBAD-2 (C), and NIRBAD-3 (D). The excitation and emission wavelengths were scanned in a solution of 10 μM of each compound in DMSO. AUF, arbitrary units of fluorescence.

**Figure 5:**
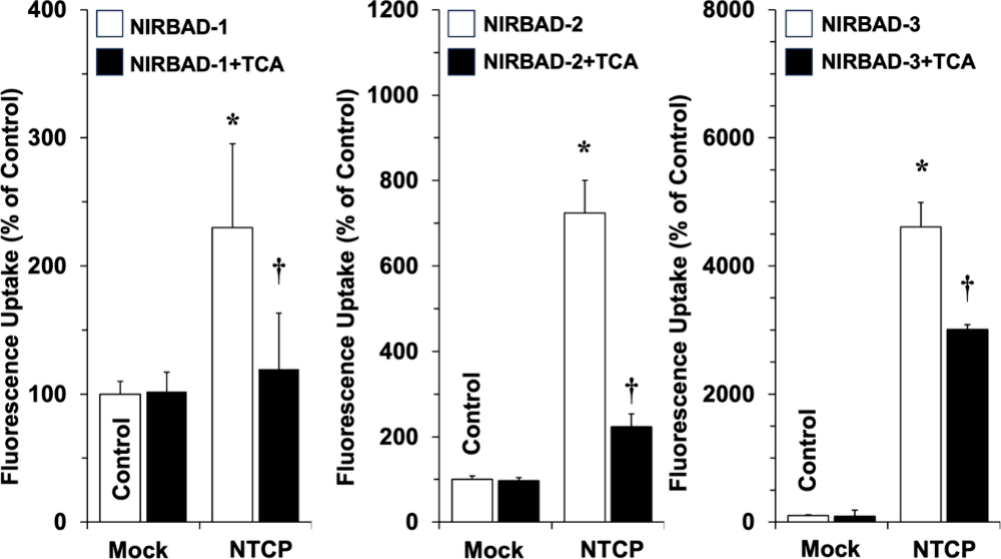
NTCP-mediated NIRBAD uptake. CHO cells transfected with empty vectors (Mock) or lentiviral particles to induce stable expression of NTCP were incubated for 15 min at 37 °C with 10 μM NIRBAD-1, NIRBAD-2, or NIRBAD-3 in the absence (white bars) or in the presence of 100 μM taurocholic acid (TCA) (black bars). Uptake was determined by flow cytometry. The detected fluorescence expressed as arbitrary units of fluorescence (AUF) per cell and 15 min were normalized in each experiment by considering 100% the value of AUF in Mock cells incubated in the absence of TCA (Control). Results are mean ± SEM from six different measurements carried out in three separate cultures. *p < 0.05; comparing with uptake by Mock cells. †*p* < 0.05; comparing with uptake in the absence of TCA (Control) in each experimental group by paired *t*-test.

The analysis of the fluorescent properties of these compounds revealed that the excitation and emission wavelengths differed from those of the initial fluorescent probe (Figure 4A). Differences among NIRBADs were also found (Figure 4B–D). Moreover, the relative quantum yields of NIRBAD-1 and NIRBAD-3 were similar but lower than that of NIRBAD-2 (Figure S13). This diversity may likely be attributed to the fact that, while the wavelengths stem from the pi-electron delocalization within the indole-alkene conjugate system, slight modifications may arise from the altered chemical environment induced by the presence of triazoles. The lower signal found for ester NIRBAD-1 as compared to that of the amide NIRBAD-3 was not due to bleaching or hydrolysis, as their photostability (Figure S14) and chemical stability (Figure S15) were similarly preserved when kept in solution at 37 °C in the dark for up to 60 min, i.e., longer than the incubation time during uptake experiments (Figure 5).

To assess the hepatocyte-targeting properties of NIRBADs, flow cytometry studies were carried out using CHO cells that either did not express any human BA transporter or stably express NTCP. The uptake of NIRBADs in the absence and presence of TCA revealed that NTCP can transport NIRBADs in a TCA-inhibitable manner (Figure 5). The order of efficacy for this transport was NIRBAD-3 ≫ NIRBAD-2 > NIRBAD-1. Based on these results, further *in vivo* assays were carried out using NIRBAD-3 as a proof of concept.

**Figure 6:**
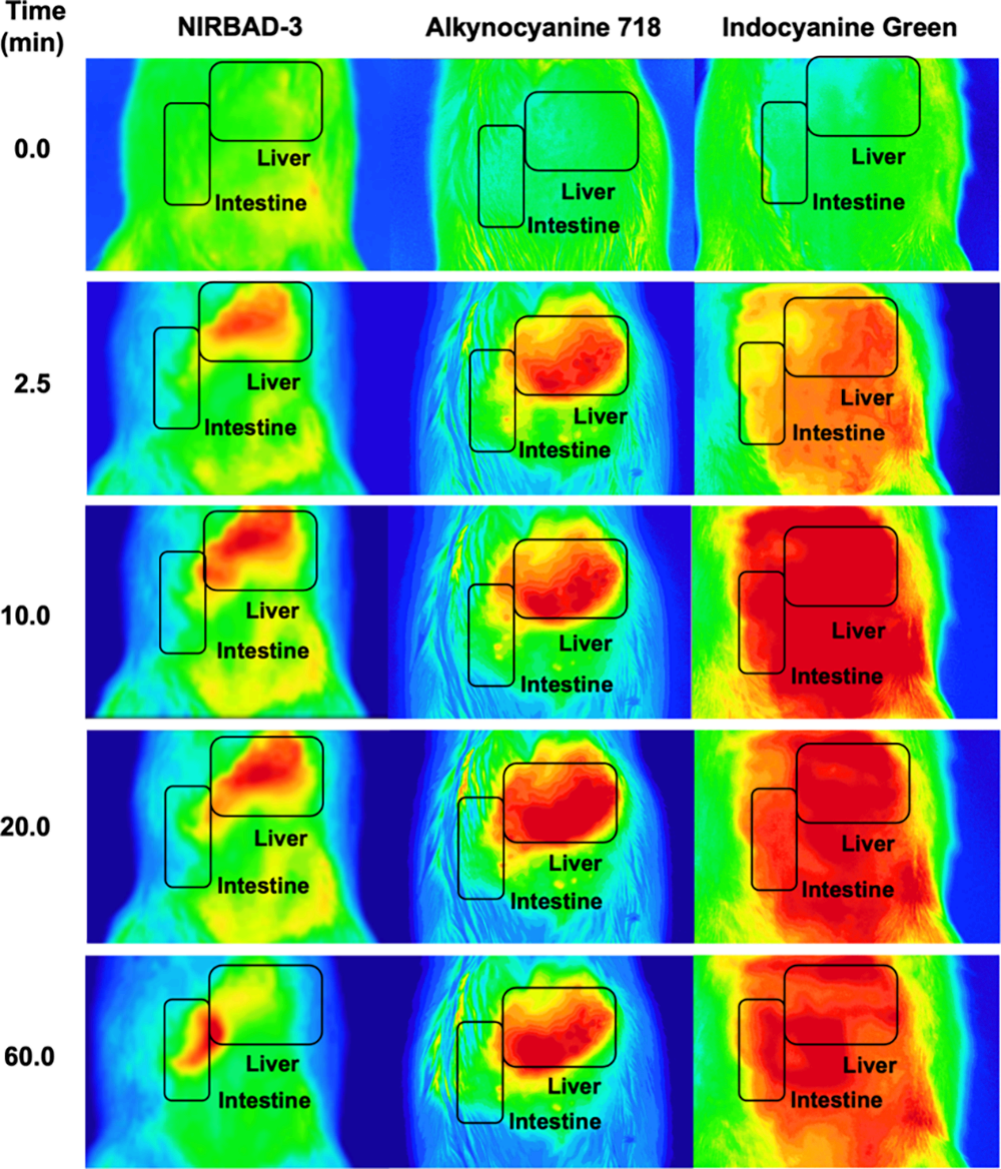
Time course of extracorporeal fluorescence detected in the abdominal area after intravenous administration of 1 μmol NIRBAD-3, alkynocyanine 718, or indocyanine green to anesthetized Wistar rats. These compounds were first dissolved in DMSO and then diluted with saline to inject 1 mL (<12% DMSO). At these doses, neither DMSO nor NIRBAD-3 caused acute liver/renal toxicity.

Upon i.v. injection of NIRBAD-3 or alkynocyanine 718, the fluorescence emitted from the upper abdominal region was extracorporeally recorded. In the case of NIRBAD-3, an efficient liver load, followed by migration of the fluorescence toward the intestine, was observed (Figure 6). The hepatic elimination was achieved at approximately 60 min. Determinations carried out in blood samples collected 120 min after NIRBAD-3 administration revealed no change in serum biomarkers of hepatic and renal toxicity (Table 1).

**Table 1:** Effect of NIRBAD-3 Administration to Rats on Serum Biochemical Markers of Hepatic and Renal Toxicity^a^

	NIRBAD-3	**reference values**
T-Pro (g/dL)	5.0 ± 0.5	5.6–7.6
Alb (g/dL)	2.7 ± 0.3	3.8–4.8
T-Bil (mg/dL)	0.4 ± 0.1	<0.6
AST (IU/L)	64.3 ± 22.2	<80.8
ALT (IU/L)	12.3 ± 1.5	<30.2
BUN (mg/dL)	22.3 ± 1.5	<21.0
UA (mg/dL)	1.3 ± 0.1	<1.4
Cre (mg/dL)	0.3 ± 0.1	<0.8

a The rats (*n* = 5) received NIRBAD-3 (1 μmol. i.v.) 120 min before blood collection. Values are mean ± SEM. T-Pro, total protein; Alb, albumin; T-Bil, total bilirubin; AST, aspartate aminotransferase; ALT, alanine aminotransferase; BUN, blood urea nitrogen; UA, uric acid; Cre, creatinine; IU, International units.

In contrast to NIRBAD-3, the fluorescence due to alkynocyanine 718 persisted in the liver with no transfer to the intestinal area. We also administered ICG for comparison purposes, whose fluorescence was dispersedly distributed in the abdominal region without showing any hepatic selectivity. Moreover, the fluorescence remained in this location throughout the 60 min experimental period (Figure 6).

In conclusion, novel BA derivatives have been synthesized. They are selectively taken up by the liver and efficiently transferred to the intestine by biliary secretion, as expected for a cholephilic substance. This organotropic characteristic, together with their ability to emit NIR fluorescence, permits extracorporeal detection for monitoring their liver handling. The usefulness of these probes in noninvasively assessing liver function in health and diseases using experimental models deserves further investigation.

## Experimental Procedures

### Chemicals

CA, taurocholic acid (TCA), diciclohexilcarbodiimide (DCC), 4-(dimethylamino)pyridine (DMAP), 2-chloroethanol, 2-chloroethanamine, ICG, sodium ascorbate, and copper(II) acetate were obtained from Sigma-Aldrich (Merck, Madrid, Spain). All these compounds ensured a high-grade purity in line with synthesis standards (≥98%). All other chemicals and organic solvents were of analytical grade. CGamF was synthesized following the published protocols.^12^ The alkynocyanine 718 (CAS: 1188292–54-7) was obtained from Luminochem Ltd., (Budapest, Hungary).

### General Chemical Procedures

Solvents were purified by standard procedures and distilled before use. Reagents and starting materials obtained from commercial suppliers were used without further purification. Melting points are given in °C. ^1^H NMR spectra were recorded on a Bruker Avance spectrophotometer at 400, 200, and 100 MHz, as appropriate. ^1^H NMR chemical shifts are reported in ppm with tetramethylsilane (TMS) as the internal standard or using the residual solvent ^1^H resonance as a reference. The coupling constants, *J*, are reported in Hertz (Hz). Data for ^1^H NMR are reported as follows: chemical shift (in ppm), number of hydrogen atoms, multiplicity (s = singlet, d = doublet, t = triplet, q = quartet, quint = quintet, m = multiplet, br s = broad singlet). Splitting patterns that could not be clearly distinguished are denoted as multiplets (m). High-resolution mass spectral analyses (HRMS) were performed using ESI ionization and a quadrupole TOF mass analyzer. All compounds were routinely checked by TLC using precoated silica gel 60 F254, aluminum foil, and the spots were detected under UV light at 254 and 365 nm or were revealed spraying with 10% phosphomolybdic acid in ethanol. Flash chromatography (FC) was performed on 70–200 mesh silica gel using a different composition of the mobile phase according to the polarity of the compound.

### Synthesis of BA Derivatives

#### 2-Chloroethyl (*R*)-4-((3*R*,5*S*,7*R*,8*R*,9*S*,10*S*,12*S*,13*R*,14*S*,17*R*)-3,7,12-trihydroxy-10,13-dimethylhexadecahydro-1*H*-cyclopenta[*a*]phenanthren-17-yl)pentanoate (**1**)

In a flask provided with a magnetic stirring bar, CA (253 mg, 0.620 mmol), DCC (128 g, 0.620 mol), DMAP (76.0 mg; 0.540 mmol), 2-chloroethanol (2.0 mL; 29.9 mmol), and chloroform (2 mL) were added. The progress of the reaction was followed by TLC and upon completion. The mixture was then extracted with chloroform and washed with 4% aqueous Na_2_CO_3_ solution. The organic phase was dried over anhydrous Na_2_SO_4_, and the solvent was distilled off under reduced pressure to afford compound **1** (274 mg, 95% yield). Spectroscopic data (Figure S1) were as follows: ^1^H NMR (400 MHz, CDCl_3_) δ (ppm): 0.67 (3H, s); 0.87 (3H, s); 0.98 (3H, d, *J* = 5,9 Hz); 1.07–2.36 (24H, m); 3.40–3.50 (1H, m); 3.67 (2H, t, *J* = 6.0 Hz); 3.79–3.86 (1H, m); 3.93–3.98 (1H, m); 4,32 (2H, t, *J* = 6.0 Hz).HRMS for C_26_H_43_ClNaO_5_ was 493.2697; found *m*/*z* was 493.2696.

#### (*R*)-*N*-(2-Chloroethyl)-4-((3*R*,5*S*,7*R*,8*R*,9*S*,10*S*,12*S*,13*R*,14*S*,17*R*)-3,7,12-trihydroxy-10,13-dimethylhexadecahydro-1*H*-cyclopenta[*a*]phenanthren-17-yl)pentanamide (**2**)

In a flask provided with a magnetic stirring bar, CA (300 mg, 0.730 mmol), DCC (151 g, 0.730 mol), DMAP (89.2 mg; 0.730 mmol), and CHCl_3_ (10 mL) were added. In another flask, 2-chloroethanamine (423 mg; 3.65 mmol), triethylamine (369 mg, 3.65 mmol), and chloroform (2 mL) were added. The second solution was added to the first, and the resulting reaction was allowed to react at room temperature and with stirring for 48 h, following its progress by TLC upon completion. The mixture was then extracted with chloroform and washed with HCl 1 M solution, followed by a NaCl-saturated dissolution. The organic phase was dried over anhydrous Na_2_SO_4_, and the solvent was distilled off under reduced pressure to isolate compound **2** (145 mg, 42% yield). Spectroscopic data (Figure S2) were as follows: ^1^H NMR (200 MHz, CD_3_OD) δ (ppm): 0.70 (3H, s); 0.90 (3H, s); 1.02 (3H, d, *J* = 5.9 Hz); 1.05–2.36 (24H, m); 3.25–3.31 (2H, sa); 3.40–3.50 (1H, m); 3.58 (2H, t, *J* = 6.0 Hz). 3.74–3.81 (1H, m); 3.90–3.97 (1H, m); 8.26 (1H, t, *J* = 4.7 Hz). HRMS for C_26_H_44_Cl_2_O_4_ was 504.2647; found *m*/*z* was 504.2653.

#### 2-Azidoethyl (*R*)-4-((3*R*,5*S*,7*R*,8*R*,9*S*,10*S*,12*S*,13*R*,14*S*,17*R*)-3,7,12-trihydroxy-10,13-dimethylhexadecahydro-1*H*-cyclopenta[*a*]phenanthren-17-yl)pentanoate (**3**)

In a flask provided with a magnetic stirring bar, compound **1** (150 mg; 0.320 mmol), sodium azide (26.0 mg; 0.400 mmol), and DMF (3 mL) were added. The reaction mixture was allowed to react at 100 °C for 48 h. The progress of the reaction was followed by TLC and upon completion. The mixture was then extracted with ethyl acetate and washed with 4% aqueous Na_2_CO_3_ solution. The organic phase was dried over anhydrous Na_2_SO_4_, and the solvent was distilled off under reduced pressure to isolate compound **3** (145 mg, 95% yield). Spectroscopic data (Figure S3) were consistent with those previously described.^25^ These were as follows: ^1^H NMR (400 MHz, CDCl_3_) δ (ppm): 0.87 (3H, s); 0.96 (3H, s); 0.98 (3H, d, *J* = 5,9 Hz); 0.98–2.46 (24H, m); 3.40–3.50 (1H, m); 3.46 (2H, t, *J* = 5.2 Hz); 3.79–3.85 (1H, m); 3.92–3.98 (1H, m); 4.23 (2H, t, *J* = 5.2 Hz); HRMS for C_26_H_43_N_3_NaO_5_ was 500.3100; found *m*/*z* was 500.3091.

#### (*R*)-*N*-(2-Azidoethyl)-4-((3*R*,5*S*,7*R*,8*R*,9*S*,10*S*,12*S*,13*R*,14*S*,17*R*)-3,7,12-trihydroxy-10,13-dimethylhexadecahydro-1*H*-cyclopenta[*a*]phenanthren-17-yl)pentanamide (**4**)

In a flask provided with a magnetic stirring bar, compound **2** (350 mg; 0.74 mmol), sodium azide (58.0 mg; 0.890 mmol), and DMF (3 mL) were added. The reaction mixture was allowed to react at 100 °C for 48 h. The progress of the reaction was followed by TLC and upon completion. The mixture was then extracted with ethyl acetate and washed with 4% aqueous Na_2_CO_3_ solution. The organic phase was dried over anhydrous Na_2_SO_4_, and the solvent was distilled off under reduced pressure to isolate compound **4** (324 mg, 92% yield). Spectroscopic data (Figure S4) were as follows: ^1^H NMR (400 MHz, CDCl_3_) δ (ppm): 0.68 (3H, s); 0.88 (3H, s); 0.99 (3H, d, *J* = 5.9 Hz); 0.98–2.46 (24H, m); 3.39–3.48 (5H, m); 3.83–3.86 (1H, m); 3.95–3.99 (1H, m); 6.35 (1H, m). HRMS for C_26_H_44_N_4_O_5_Na was 499,3260; found *m*/*z* was 499,3248.

#### 4-(2-((*E*)-2-((*E*)-3-(2-((*E*)-3,3-Dimethyl-1-(4-sulfobutyl)indolin-2-ylidene)ethylidene)-2-(((1-(2-(((*R*)-4-((3*R*,5*S*,7*R*,8*R*,9*S*,10*S*,12*S*,13*R*,14*S*,17*R*)-3,7,12-trihydroxy-10,13-dimethylhexadecahydro-1*H*-cyclopenta[a]phenanthren-17-yl)pentanoyl)oxy)ethyl)-1*H*-1,2,3-triazol-4-yl)methyl)amino)cyclopent-1-en-1-yl)vinyl)-3,3-dimethyl-3*H*-indol-1-ium-1-yl)butane-1-sulfonate (**5**, NIRBAD-1)

Compound **3** (58.7 mg; 0.123 mmol) was added to a mixture of sodium ascorbate (24.0 mg; 0.123 mmol), copper(II) acetate (6.0 mg; 0.033 mmol), alkynocyanine 718 (60.0 mg; 0.082, mmol), and 5 mL of ethanol. The mixture was left to react at room temperature, with stirring, in an argon atmosphere, and in darkness. After 7 days, the reaction was complete. The crude product was isolated by PTLC chromatography over SiO_2_ using a 65:25:15:5 mixture of CHCl_3_/MeOH/CH_3_–COOH/H_2_O as the eluent, and 15 mg of the pure compound NIRBAD-1 (15% yield) was obtained. Mp: decomposition at 150 °C. Spectroscopic data (Figure S5) were as follows: ^1^H NMR (400 MHz, DMSO-d_6_) δ (ppm): 0.52 (3H, s); 0.78 (3H, s); 0.84 (3H, d, *J* = 6.1 Hz); 1.22 (8H, s); 1.41 (12H, s) 0.80–2.40 (24H, m); 2.72 (2H, s); 3.12–3.17 (5H, m); 3.40–3.55 (1H, m); 3.57 (1H, m); 3.72 (2H, sa); 3.88 (4H, m); 3.88–4.37 (2H, m); 4.37 (2H, t, *J* = 4.7); 4.64 (2H, t, *J* = 4.7 Hz); 5.01 (2H, m); 5.58 (2H, d, *J* = 12.2 Hz); 6.99 (2H, t, *J* = 7.5 Hz); 7.09 (2H, d, *J* = 7.9 Hz), 7.24 (2H, t, *J* = 7.7 Hz); 7.36 (2H, d, *J* = 7.2 Hz); 7.74 (2H, d, *J* = 12.2 Hz); 8.18 (1H, s). HRMS for C_66_H_91_N_6_O_11_S_2_ was 1207.6187; found *m*/*z* was 1207.3202.

#### 4-(2-((*E*)-2-((*E*)-3-(2-((*E*)-3,3-Dimethyl-1-(4-sulfobutyl)indolin-2-ylidene)ethylidene)-2-(((1-(2-((*R*)-4-((3*R*,5*S*,7*R*,8*R*,9*S*,10*S*,12*S*,13*R*,14*S*,17*R*)-3,7,12-trihydroxy-10,13-dimethylhexadecahydro-1*H*-cyclopenta[a]phenanthren-17-yl)pentanamido)ethyl)-1*H*-1,2,3-triazol-4-yl)methyl)amino)cyclopent-1-en-1-yl)vinyl)-3,3-dimethyl-3*H*-indol-1-ium-1-yl)butane-1-sulfonate (**6**, NIRBAD-3)

Compound **4** (25.4 mg; 0.050 mmol) was added to a mixture of sodium ascorbate (8.1 mg; 0.042 mmol), copper(II) acetate (0.8 mg; 0.005 mmol), alkynocyanine 718 (30.0 mg; 0.004, mmol), and 5 mL of ethanol. The mixture was left to react at room temperature, with stirring, in an argon atmosphere and in complete darkness. After 7 days, the reaction was complete. The crude product was isolated by PTLC chromatography over SiO_2_ using a 65:25:15:5 mixture of CHCl_3_/MeOH/CH_3_–COOH/H_2_O as the eluent, and 12 mg of the pure compound NIRBAD-3 (20% yield) was obtained. Spectroscopic data (Figure S6) were as follows: Mp decomposition at 130 °C. ^1^H NMR (400 MHz, DMSO-d_6_) δ (ppm): 0.52 (3H, s); 0.78 (3H, s); 0.84 (3H, d, *J* = 6.1 Hz); 1.22 (8H, s); 1.41 (12H, s) 0.80–2.40 (24H, m); 2.72 (2H, s); 3.12–3.17 (5H, m); 3.40–3.55 (1H, m); 3.57 (1H, m); 3.72 (2H, sa); 3.88 (4H, m); 3.88–4.37 (2H, m); 4.37 (2H, t, *J* = 4.7); 4.64 (2H, t, *J* = 4.7 Hz); 5.01 (2H, m); 6.01 (2H, d, *J* = 12.2 Hz); 6.94 (2H, t, *J* = 7.5 Hz); 7.09 (2H, d, *J* = 7.9 Hz), 7.21 (2H, t, *J* = 7.7 Hz); 7.32 (2H, d, *J* = 7.2 Hz); 7.64 (2H, sa); 8.05 (1H, s). HRMS for C_66_H_92_N_7_O_10_S_2_ was 1206.6347; found *m*/*z* was 1206.6359.

#### Methyl (*R*)-4-((3*R*,5*S*,7*R*,8*R*,9*S*,10*S*,12*S*,13*R*,14*S*,17*R*)-3,7,12-trihydroxy-10,13-dimethylhexadecahydro-1*H*-cyclopenta[*a*]phenanthren-17-yl)pentanoate (**7**)

To a well-stirred solution of CA (1.0 g, 2.44 mmol) in methanol (10 mL), a thionyl chloride (5 mL, 68.7 mmol) was slowly added. Once the addition was complete, the reaction mixture was kept under stirring at 70 °C for half an hour. The solvent was then evaporated under reduced pressure. Compound **7** was obtained (724 mg, 70% yield). Spectroscopic data (Figure S7) were consistent with those previously described.^26^ These were as follows: ^1^H NMR (200 MHz, CDCl_3_) δ (ppm): 0.68 (3H, s); 0.88 (3H, s); 0.97 (3H, d, *J* = 5.9 Hz); 0.79–2.34 (24H, m); 3.43–3.45 (1H, m); 3.66 (3H, s); 3.83–3.87 (1H, m); 3.95–3.99 (1H, m).

#### Methyl (*R*)-4-((3*R*,5*R*,7*R*,8*R*,9*S*,10*S*,12*S*,13*R*,14*S*,17*R*)-7,12-dihydroxy-10,13-dimethyl-3-tosylhexadecahydro-1*H*-cyclopenta[*a*]phenanthren-17-yl)pentanoate (**8**)

In a flask provided with a magnetic stirring bar, compound **7** (603 mg, 1.43 mmol), tosyl chloride (272 mg, 1.43 mmol), and pyridine (5 mL) were added and the resulting mixture was left with stirring at room temperature for 24 h. The progress of the reaction was followed by TLC and upon completion. The crude mixture was dissolved in ethyl acetate. The organic phase was washed with 4% aqueous sodium carbonate solution and dried over anhydrous Na_2_SO_4_, and the solvent was evaporated under reduced pressure. The crude product was isolated by chromatography over SiO_2_ using a 9:1 mixture of CH_2_Cl_2_ and MeOH as the eluent, and 304 mg of the pure compound **8** (37% yield) was isolated. Spectroscopic data (Figure S8) were consistent with those previously described.^27^ These were as follows: ^1^H NMR (200 MHz, CDCl_3_) δ (ppm): 0.65 (3H, s); 0.84 (3H, s); 0.95 (3H, d, *J* = 5.9 Hz); 0.79–2.54 (24H, m); 2.42 (3H, s); 3.65 (3H, s); 3.82–3.83 (1H, m); 3.91–3.95 (1H, m). 3.29–3.33 (1H, m); 7.30 (2H, d, *J* = 8.2 Hz); 7.76 (2H, d, *J* = 8.2 Hz)

#### Methyl (*R*)-4-((3*S*,5*R*,7*R*,8*R*,9*S*,10*S*,12*S*,13*R*,14*S*,17*R*)-7,12-dihydroxy-3-iodo-10,13-dimethylhexadecahydro-1*H*-cyclopenta[*a*]phenanthren-17-yl)pentanoate (**9**)

In a flask provided with a magnetic stirring bar, compound **8** (500 mg, 0.87 mmol), potassium iodide (2.88 g, 17.35 mmol), and DMF (7 mL) were added and the reaction mixture was kept under stirring at 90 °C for 2 h, following the progress of the reaction by NMR. The crude mixture was dissolved in ethyl acetate. The organic phase was washed with aqueous sodium chloride solution and dried over anhydrous Na_2_SO_4_, and the solvent was evaporated under reduced pressure. Compound **9** was obtained (337 mg, 81.6% yield). Spectroscopic data (Figure S9) were consistent with those previously described.^28^ These were as follows: ^1^H NMR (200 MHz, CDCl_3_) δ (ppm): 0.67 (3H, s); 0.85 (3H, s); 0.96 (3H, d, *J* = 5.9 Hz); 0.79–2.54 (24H, m); 3.65 (3H, s); 3.81–3.85 (1H, m); 3.97–4.01 (2H, m).

#### Methyl (*R*)-4-((3*R*,5*S*,7*R*,8*R*,9*S*,10*S*,12*S*,13*R*,14*S*,17*R*)-3-Azido-7,12-dihydroxy-10,13-dimethylhexadecahydro-1*H*-cyclopenta[*a*]phenanthren-17-yl)pentanoate (**10**)

In a flask provided with a magnetic stirring bar, compound **9** (290 mg; 0.54 mmol), sodium azide (53.0 mg; 0.82 mmol), and DMF (5 mL) were added. The reaction mixture was allowed to react at 60 °C for 24 h. The progress of the reaction was followed by TLC and upon completion. The mixture was then extracted with ethyl acetate and washed with 4% aqueous Na_2_CO_3_ solution. The organic phase was dried over anhydrous Na_2_SO_4_, and the solvent was distilled off under reduced pressure to isolate compound **10** (187 mg, 76.8% yield). Spectroscopic data (Figure S10) were consistent with those previously described.^29^ These were as follows: ^1^H NMR (200 MHz, CDCl_3_) δ (ppm): 0.68 (3H, s); 0.91 (3H, s); 0.96 (3H, d, *J* = 5.9 Hz); 0.79–2.54 (24H, m); 3.65 (3H, s); 3.82–3.89 (2H, m); 3.95–3.99 (1H, m).

#### (*R*)-4-((3*R*,5*S*,7*R*,8*R*,9*S*,10*S*,12*S*,13*R*,14*S*,17*R*)-3-Azido-7,12-dihydroxy-10,13-dimethylhexadecahydro-1*H*-cyclopenta[*a*]phenanthren-17-yl)pentanoic acid (**11**)

In a flask provided with a magnetic stirring bar, compound **10** (187 mg, 0.42 mmol), was added to a solution of LiOH in methanol (10 mL, 2M). The resulting mixture was left under stirring at room temperature for 24 h. The progress of the reaction was followed by NMR. The solvent was evaporated under reduced pressure, and 2 M HCl was added. The resulting mixture was dissolved in ethyl acetate. The organic layer was dried over anhydrous Na_2_SO_4_. The solvent was distilled off under reduced pressure to isolate compound **11** (128 mg, 70.5% yield). Spectroscopic data (Figure S11) were consistent with those previously described.^30^ These were as follows: ^1^H NMR (200 MHz, CDCl_3_) δ (ppm): 0.70 (3H, s); 0.91 (3H, s); 1.00 (3H, d, *J* = 5.9 Hz); 0.79–2.54 (24H, m); 3.64 (1H, m); 3.77–3.81 (1H, m); 3.93–3.96 (1H, m).

#### 4-(2-((*E*)-2-((*E*)-2-(((1-((3*R*,5*S*,7*R*,8*R*,9*S*,10*S*,12*S*,13*R*,14*S*,17*R*)-17-((*R*)-4-Carboxybutan-2-yl)-7,12-dihydroxy-10,13-dimethylhexadecahydro-1*H*-cyclopenta[*a*]phenanthren-3-yl)-1*H*-1,2,3-triazol-4-yl)methyl)amino)-3-(2-((*E*)-3,3-dimethyl-1-(4-sulfobutyl)indolin-2-ylidene)ethylidene)cyclopent-1-en-1-yl)vinyl)-3,3-dimethyl-3*H*-indol-1-ium-1-yl)butane-1-sulfonate (**12**, NIRBAD-2)

Compound **11** (23 mg; 0.053 mmol) was added to a mixture of sodium ascorbate (8.4 mg; 0.042 mmol), copper(II) acetate (1.4 mg; 0.007 mmol), alkynocyanine 718 (30.0 mg; 0.041, mmol), and 7 mL of ethanol. The mixture was left to react at room temperature, with stirring, in an argon atmosphere and in complete darkness. After 7 days, the reaction was complete. The crude product was isolated by PTLC chromatography over SiO_2_ using a 65:20:15:5 mixture of CHCl_3_/MeOH/CH_3_–COOH/H_2_O as the eluent, and 12.3 mg of the pure compound NIRBAD-2 (20% yield) was obtained. Spectroscopic data (Figure S12) were as follows: Mp decomposition at 150 °C. ^1^H NMR (400 MHz, DMSO-d_6_) δ (ppm): 0.58 (3H, s); 0.82 (3H, s); 0.85 (3H, d, *J* = 6.1 Hz); 1.22 (8H, s); 1.84 (12H, s) 0.80–2.40 (27H, m); 2.74 (2H, sa); 3.05 (1H, m); 3.30–3.45 (1H, m); 3.57–3.66 (2H, m); 3.91 (4H, sa); 4.16–4.09 (2H, m); 4.27–4.34 (1H, m); 5.30–5.32 (3H, m); 5.63 (2H, d, *J* = 12.2 Hz); 6.65 (2H, sa); 7.02 (2H, t, *J* = 7.5 Hz); 7.13 (2H, d, *J* = 7.9 Hz), 7.26 (2H, t, *J* = 7.7 Hz); 7.40 (2H, d, *J* = 7.2 Hz); 7.68 (1H, m); 7.78 (2H, d, *J* = 12.2 Hz); 8.10 (1H, s). HRMS for C_64_H_87_N_6_O_10_S_2_ was 1163.5931; found *m*/*z* was 1163.5918.

### Spectrofluorimetric Studies

Absorbance and fluorescence studies were carried out with a spectrophotometer. Hitachi U-2000 and Hitachi F-4500 (Triad Scientific, Manasquan, New Jersey) with quartz cuvettes (Hellma) with a 10 mm optical path were used. The Hitachi F-4500 contained an R3788 Photomultiplier tube detector with a measurement range between 200 and 750 nm, and a light source of a 150 W Xe lamp was used. The software NI-488.2 was applied to analyze the obtained data.

### Cell Culture

The Chinese ovary hamster cell line, CHO-K1 (ATCC CCL-61), was purchased from the American Type Culture Collection (LGC Standards, Barcelona, Spain) and maintained in DMEM high-glucose medium (Merck, Madrid, Spain) supplemented with 50 g/L of l-proline (Merck), GlutaMAX solution (Fisher Scientific), 10% heat-inactivated fetal bovine serum (FBS), and 1% penicillin–streptomycin–amphotericin B (Fisher Scientific). Cells were cultured at 37 °C in a 5% CO_2_ atmosphere with 80% relative humidity. To ensure the absence of mycoplasma contamination in the culture, periodic PCR tests were conducted using the mycoplasma Gel Form Kit (Biotools B&M Laboratories, Madrid, Spain). Monoclonal cells stably expressing the ORF of NTCP were obtained by transduction with recombinant lentiviral vectors (pWPI) added to target cells at an appropriate multiplicity of infection (MOI = 25) in the presence of hexadimethrine bromide (Polybrene, Merck) as described elsewhere.^31^ The clone with the highest capacity to carry out the uptake of CGamF, a fluorescent NTCP substrate,^32^ was used in further studies.

### Flow Cytometry Determinations

Both wild-type (WT) CHO cells (transduced with pWPI empty vectors), used here as a control (Mock), and cells stably expressing NTCP (CHO-NTCP) were cultured according to previously established methods.^33^ To assess drug uptake, experiments were carried out using three to five different cultures for each data point. Subconfluent cultures were resuspended in uptake medium (96 mM NaCl, 0.8 mM MgSO_4_, 5.3 mM KCl, 1.1 mM KH_2_PO_4_, 1.8 mM CaCl_2_, 11 mM d-glucose, and 10 mM HEPES/Tris, pH 7.4). The cells were then incubated in the presence of 10 μM NIRBADs with or without 100 μM TCA at 37 °C for 15 min. Uptake was stopped by rinsing the culture dishes with 0.9 mL of ice-cold buffer, and the intracellular fluorescence was measured with a flow cytometer (FACSCalibur, Becton Dickinson, Madrid).

### *In Vivo* Assays

Male Wistar rats weighing 220–250 g were obtained from the Animal House, University of Salamanca, Spain. They were housed under controlled environmental conditions of temperature (20 °C) and light (12 h/12 h light/dark cycle). They had free access to water and standard rodent chow (Panlab, Madrid, Spain). All experimental methods adhered to ethical guidelines and regulations, with protocols approved by the University of Salamanca Ethical Committee for Laboratory Animals, after confirming that they complied with ethical approval from the Spain Ministry of Health and followed European guidelines for the care and use of laboratory animals, in accordance with the NIH Guide for the Care and Use of Laboratory Animals. All experiments were conducted under pentobarbital anesthesia (50 mg/kg b.wt., i.p., Nembutal N.R.; Abbot, Madrid), which was also used for euthanizing the animals at the end of the experiments.

NIRBADs, alkynocyanine 718, or ICG (1 μmol) were dissolved in DMSO, diluted in saline solution, and administered (i.v.) to anesthetized rats. Extracorporeal fluorescence in the upper abdominal area was tracked using a high-sensitivity (3.2 MP) CCD camera (Luminescent Image Analyzer LAS-4000 imaging system, Fujifilm Life Sciences, Madrid). Serum markers of hepatic and renal toxicity were analyzed using appropriate test strips (Spotchem II Liver-1 and Spotchem II Kidney-3, ARKRAY Factory, A. Menarini Diagnostics, Badalona, Spain) read in an automatic dry chemistry analyzer (SPOTCHEM EZ SP-4430), which determined serum levels of aspartate aminotransferase (GOT or AST, IU/dL), alanine aminotransferase (GPT or ALT, IU/dL), albumin (Alb, g/dL), bilirubin (T-Bil, mg/dL), total protein (T-Pro, g/dL), BUN (blood urea nitrogen, mg/dL), uric acid (UA, mg/dL), and creatinine (Cre, mg/dL).

### Statistical Analysis

*Post hoc* analyses, such as paired or unpaired Student *t* tests, were applied appropriately to calculate the statistical significance of differences among groups. Differences were considered significant when *p* < 0.05. Microsoft Excel (version 15.32) and GraphPad (Prism5) were used for these purposes.

## Supporting Information Available

^1^H NMR and HRMS spectra of all synthesized compounds; photostability analysis of NIRBAD-1, NIRBAD-2, and NIRBAD-3; relative quantum yield; and chemical stability (PDF)

## Abbreviations

BA: bile acid
BAD: bile acid derivative
CA: cholic acid
CHO: Chinese hamster ovary cells
CGamF: cholyl-glycyl-amido fluorescein
FBS: fetal bovine serum
ICG: indocyanine green
NIR: near-infrared radiation
NIRBAD: near-infrared radiation bile acid derivative
NTCP: Na^+^-taurocholate cotransporting polypeptide
SLC: solute carrier
TCA: taurocholic acid
