# Supplementary material for: Synthesis, Characterization, and Potential Usefulness in Liver Function Assessment of Novel Bile Acid Derivatives with Near-Infrared Fluorescence (NIRBAD)
Source: Bioconjug Chem. 2024 Jul 3;35(7):971–80. doi: 10.1021/acs.bioconjchem.4c00168 (PMC11261600; doi:10.1021/acs.bioconjchem.4c00168)
Supplement: Supplementary file 1 — bc4c00168_si_001.pdf [file bc4c00168_si_001.pdf]

Synthesis, characterization, and potential usefulness in liver function assessment of novel bile acid derivatives with near-infrared fluorescence (NIRBAD)

Alvaro G. Temprano[1#], Beatriz Sanchez de Blas[1,2#], Concepción Perez-Melero[3], Ricardo Espinosa-Escudero[1], Oscar Briz[1,2], Paula Cinca-Fernando[1], Lucía Llera[1], Maria J. Monte[1,2], Francisco A. Bermejo-Gonzalez[4], Jose J.G. Marin[1,2,*] and Marta R. Romero [1,2,*]

# Both authors contributed equally as first authors to this work.
* Both authors contributed equally as senior authors to this work

Affiliations:

[1] Experimental Hepatology and Drug Targeting (HEVEPHARM), University of Salamanca, IBSAL, 37007-Salamanca, Spain.
[2] Center for the Study of Liver and Gastrointestinal Diseases (CIBEREHD), Carlos III National Institute of Health, 28029-Madrid, Spain.
[3] Pharmaceutical Chemistry Laboratory, Pharmaceutical Sciences Department, University of Salamanca, IBSAL, 37007-Salamanca, Spain.
[4] Organic Chemistry, School of Chemistry, University of Salamanca, 37007-Salamanca, Spain.

Corresponding Author:

Jose J.G. Marin
Department of Physiology and Pharmacology
University of Salamanca
Campus Miguel de Unamuno, E.D.-231
37007-Salamanca, Spain
Telephone: 34-663182872
E-mail: jjgmarin@usal.es

Figure S1. ^{1}H-NMR and HRMS spectra of compound 1

^{1}H-NMR (400 MHz, $CDCl_3$) spectrum of compound **1**.

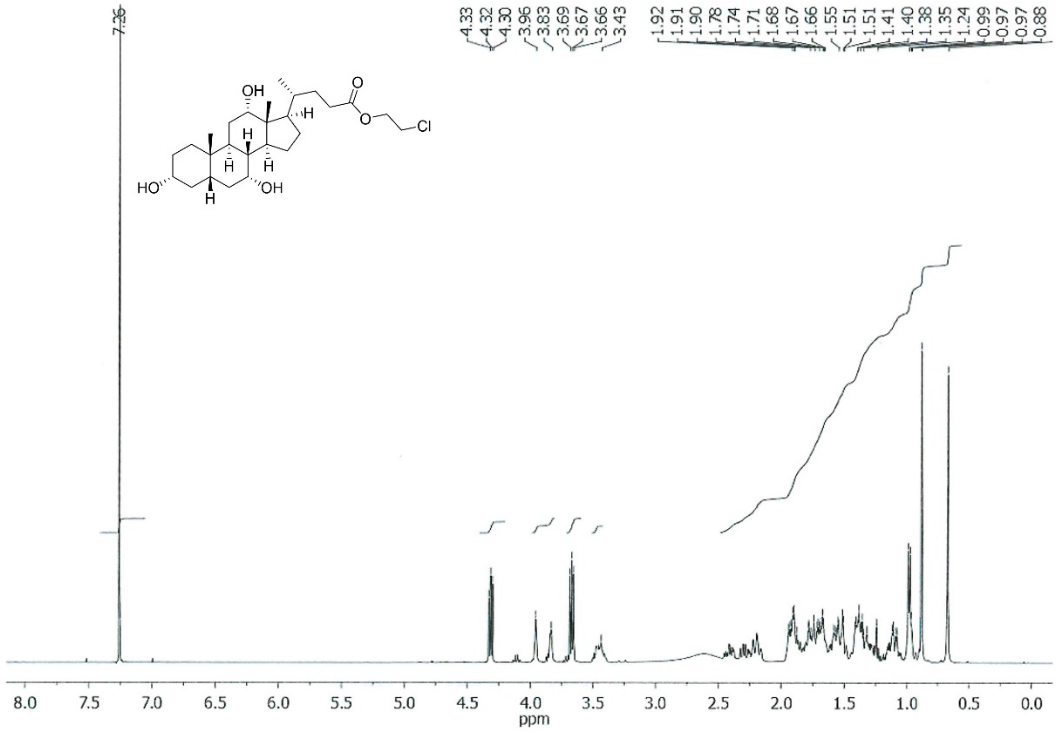

HRMS spectrum of compound **1**.

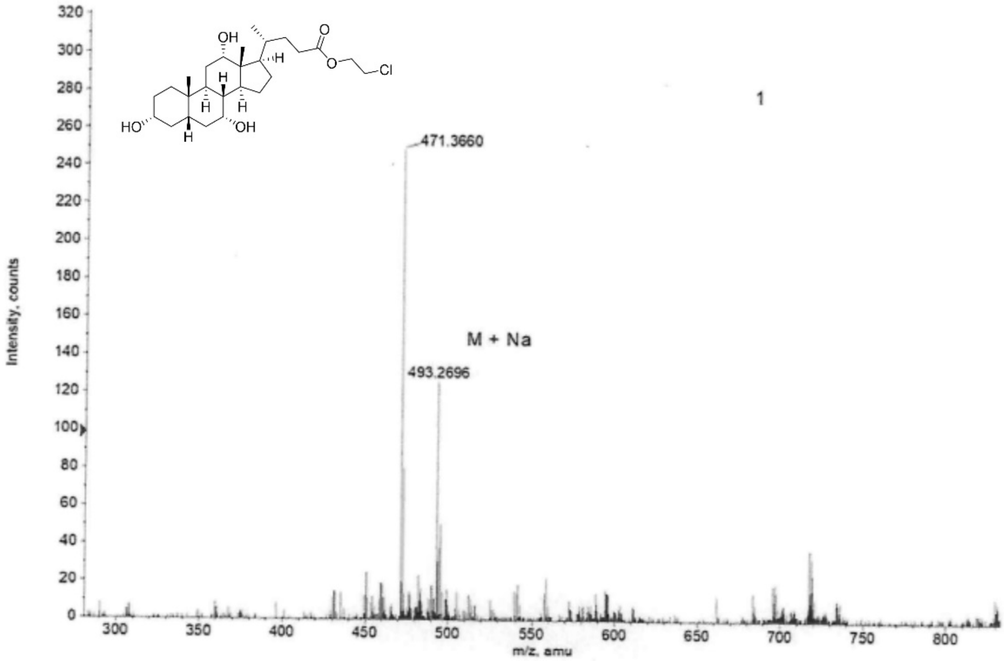

Figure S2. ^{1}H-NMR and HRMS spectra of compound 2

^{1}H-NMR (200 MHz, CD_3OD) compound **2**.

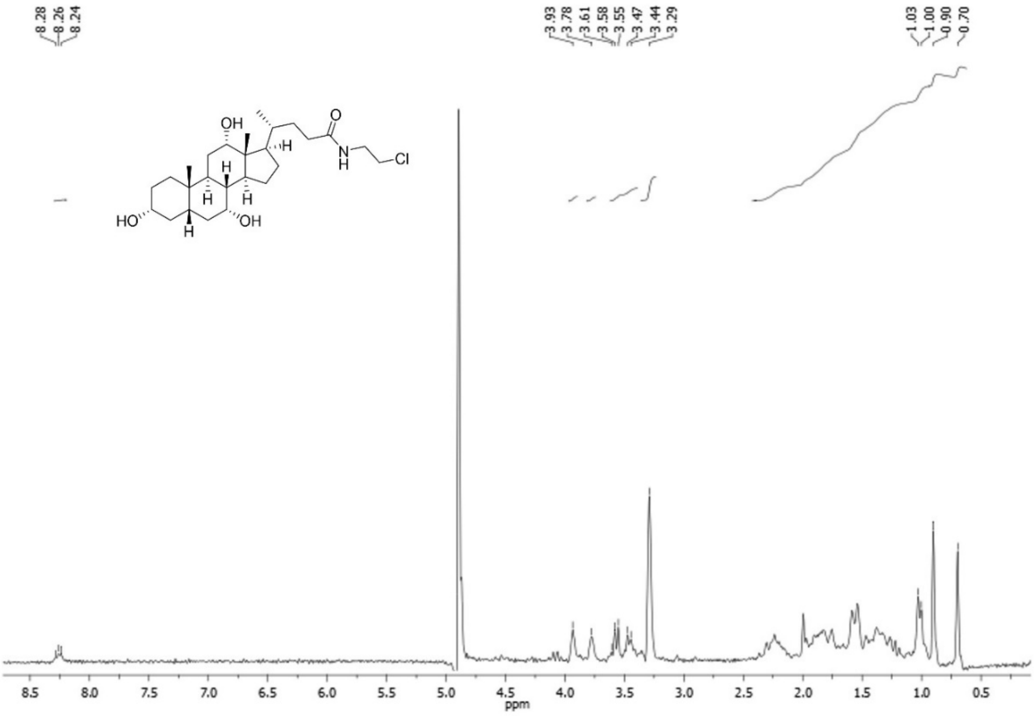

HRMS spectrum of compound **2**.

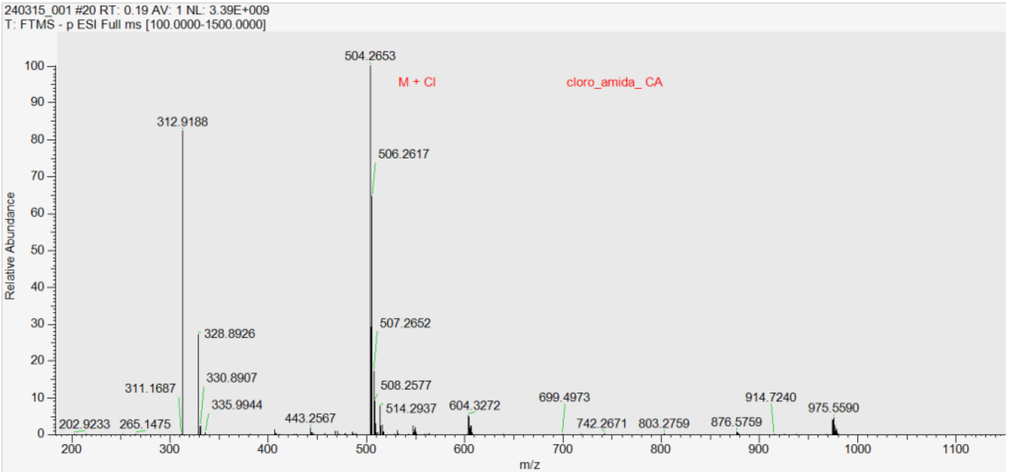

Figure S3. ^{1}H-NMR compound 3

^{1}H-NMR (400 MHz, $CDCl_3$) compound **3**.

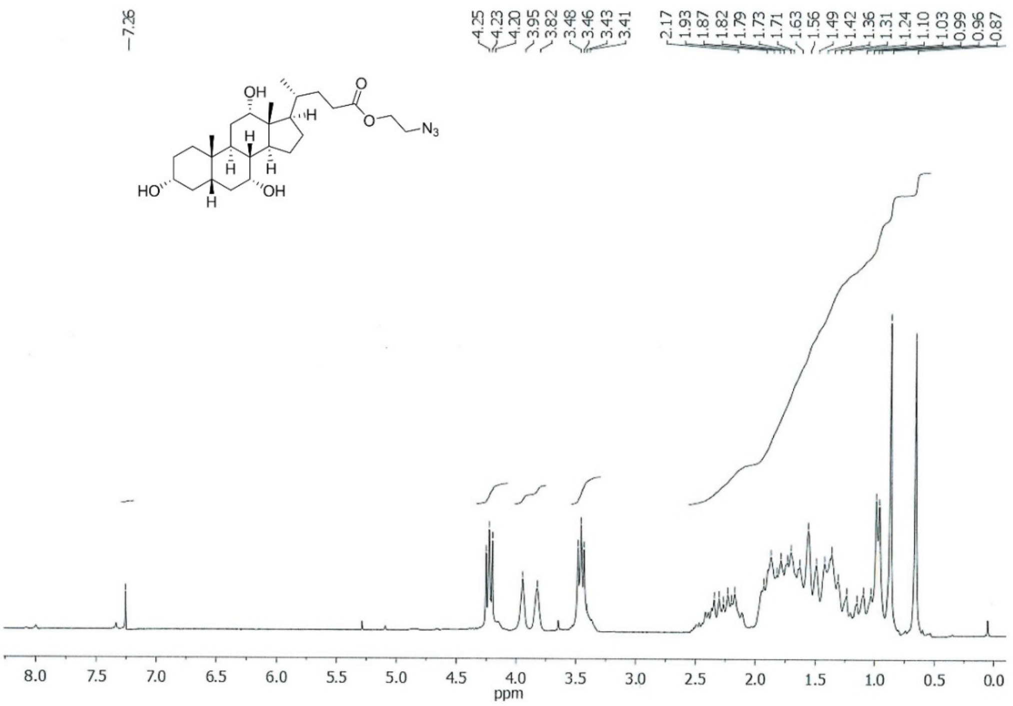

Figure S4. ¹H-NMR and HRMS spectra of compound 4

¹H-NMR (400 MHz, $CDCl_3$) compound **4**.

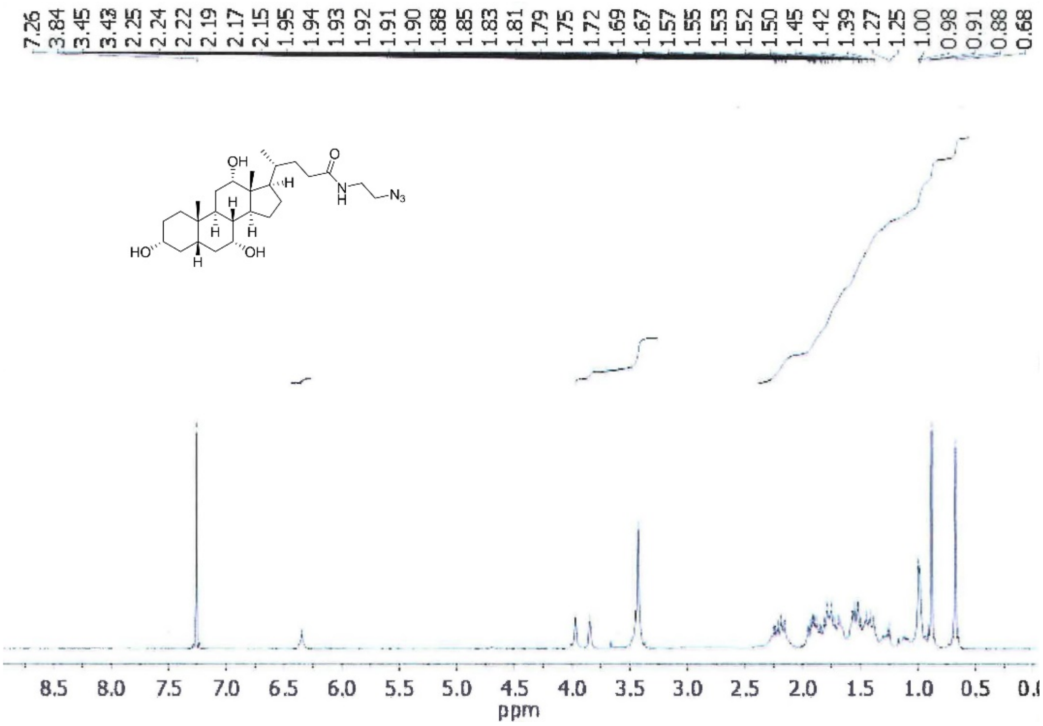

HRMS spectrum of compound **4**.

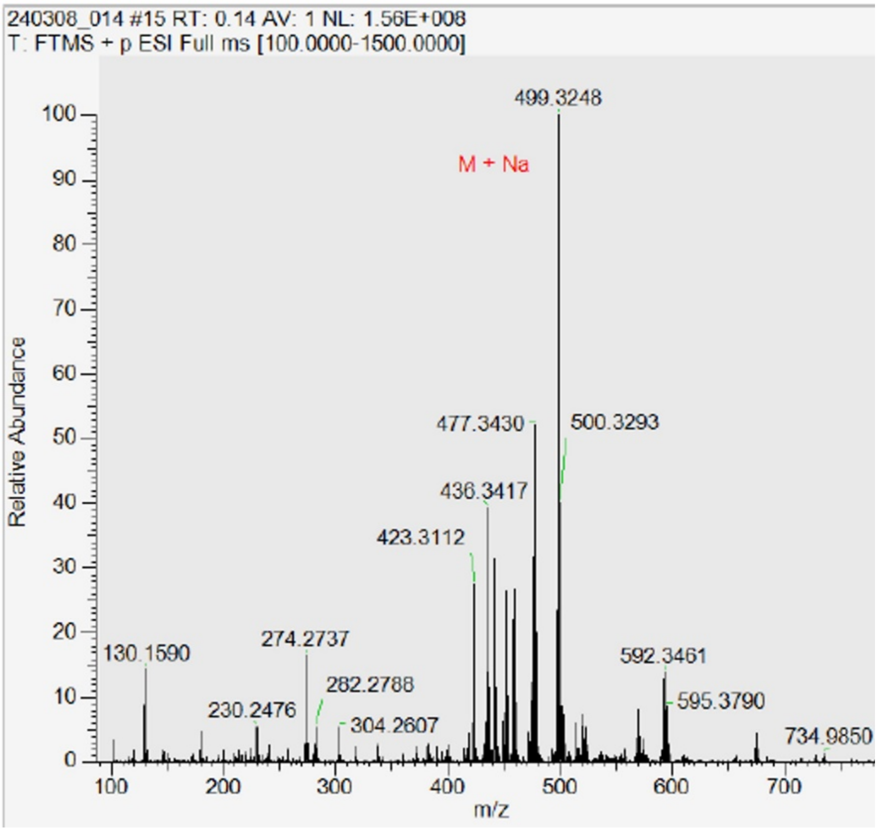

Figure S5. ^{1}H-NMR and HRMS spectra of compound 5

^{1}H-NMR (400 MHz, DMSO-d6) compound **5 NIRBAD-1**.

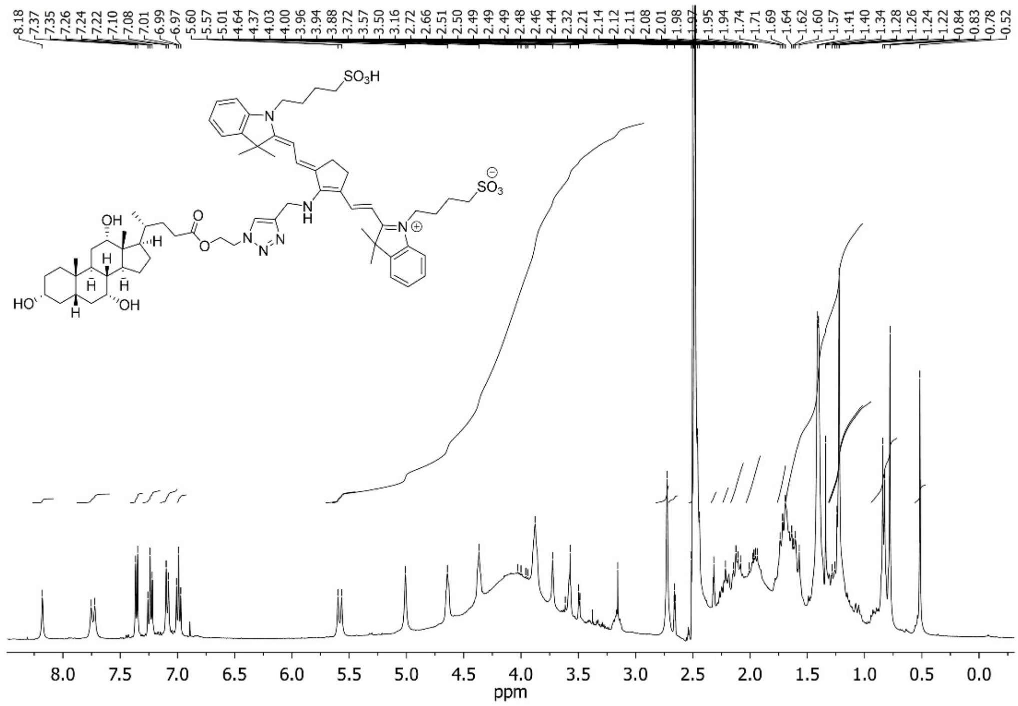

^{1}H-NMR (400 MHz, DMSO-d6) compound **5 NIRBAD-1**.

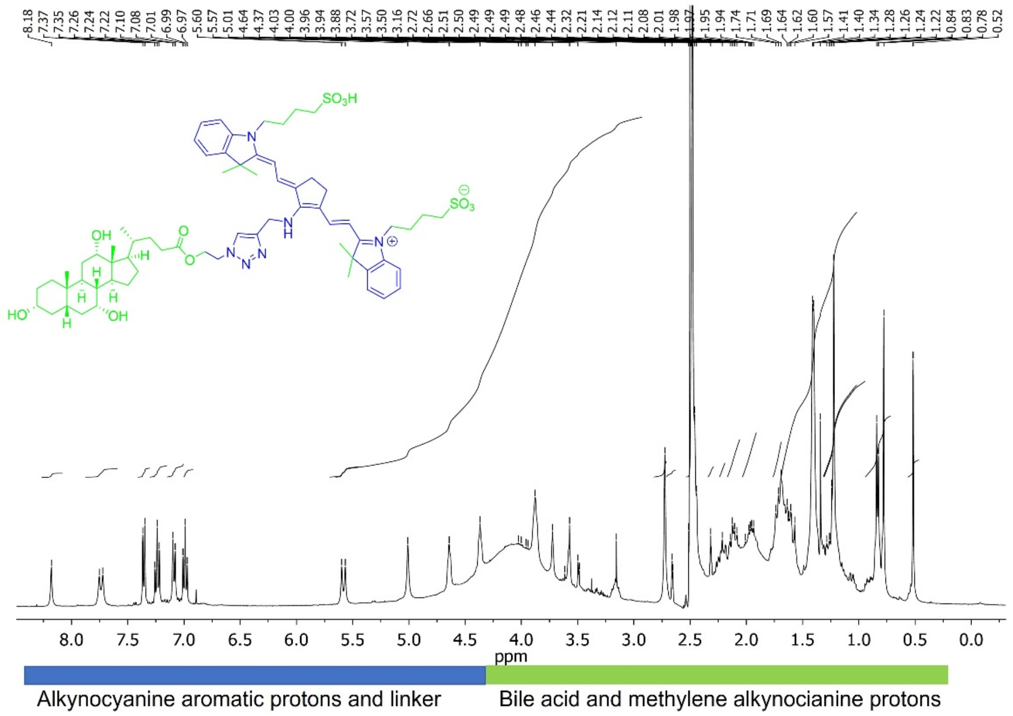

HRMS spectrum of compound **5 NIRBAD-1**.

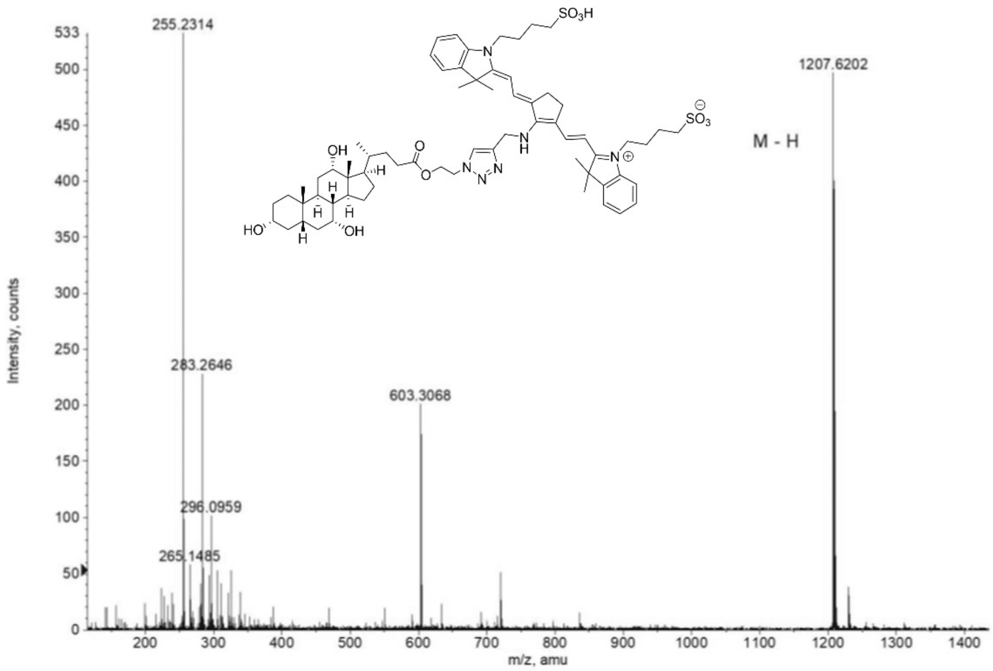

Figure S6. ^{1}H-NMR and HRMS spectra of compound 6

^{1}H-NMR (400 MHz, DMSO-d$_6$) compound **6 NIRBAD-3**.

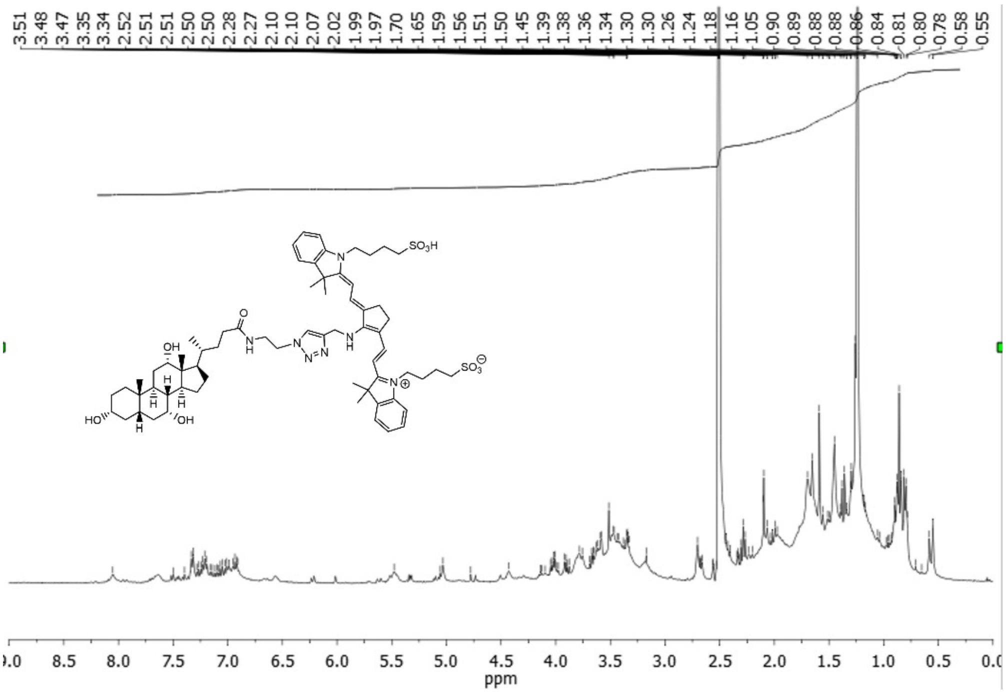

^{1}H-NMR (400 MHz, DMSO-d$_6$) compound **6 NIRBAD-3**.

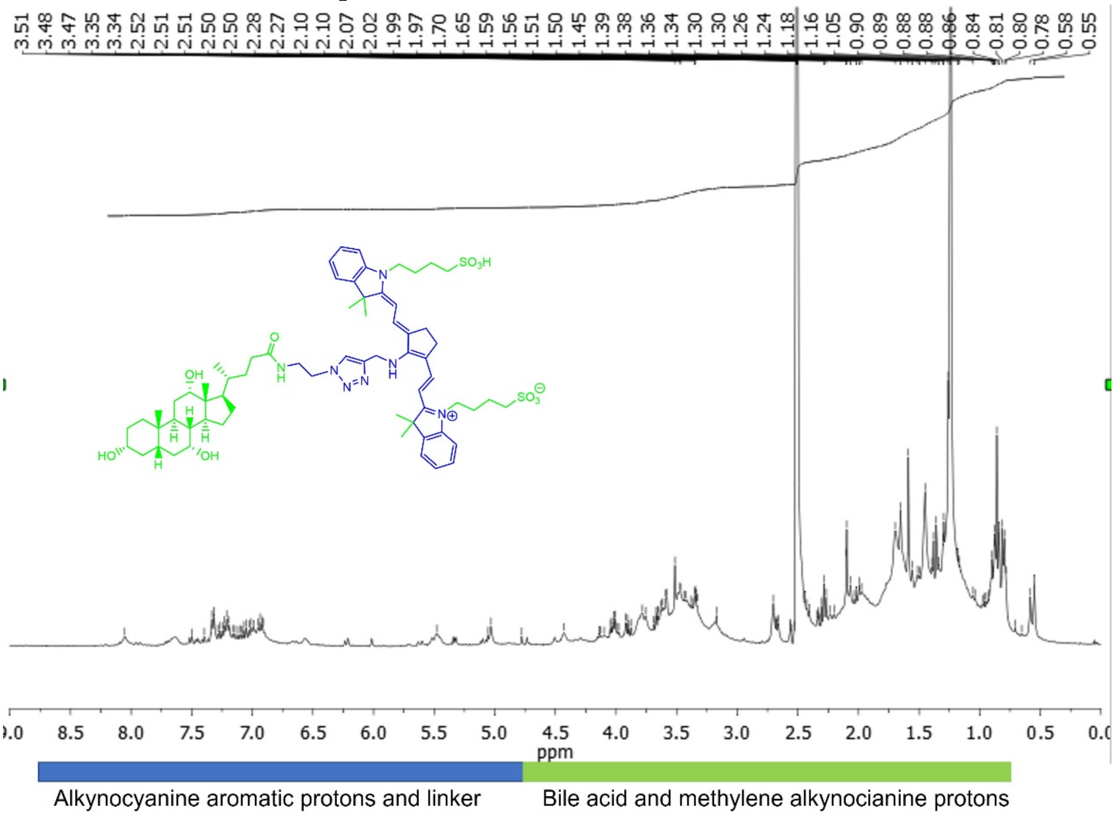

HRMS spectrum of compound **6 NIRBAD-3**

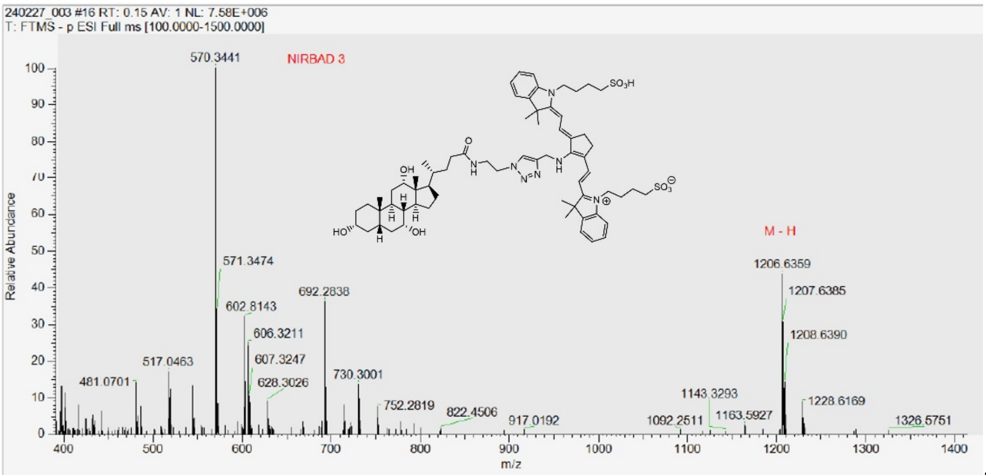

.

Figure S7. ¹H-NMR compound 7

¹H-NMR (200 MHz, $CDCl_3$) compound **7**.

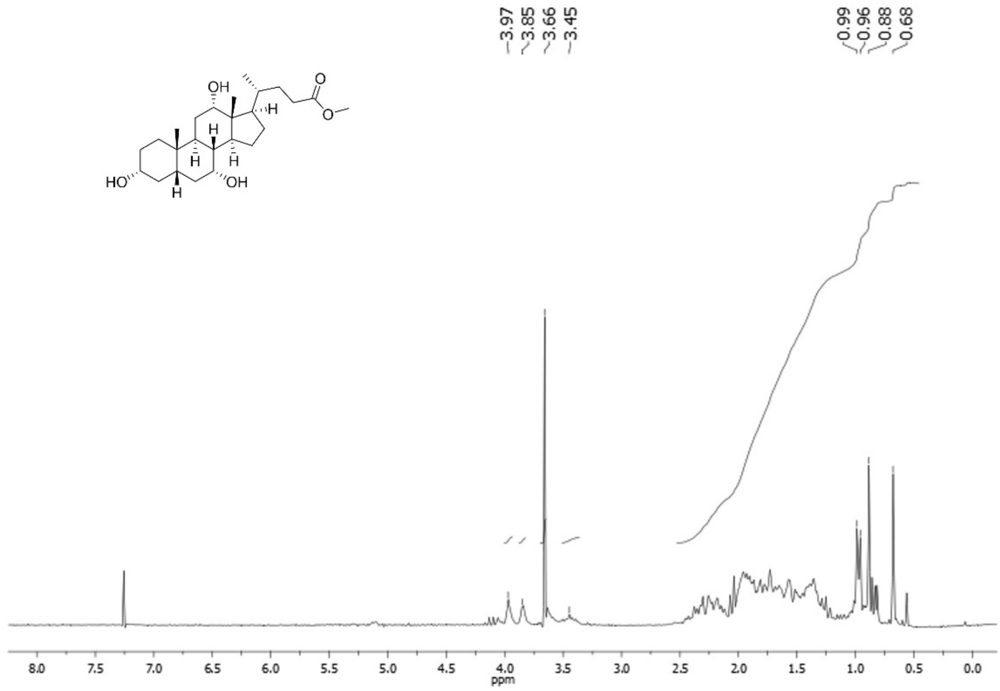

Figure S8. ¹H-NMR compound 8

¹H-NMR (200 MHz, $CDCl_3$) compound **8**.

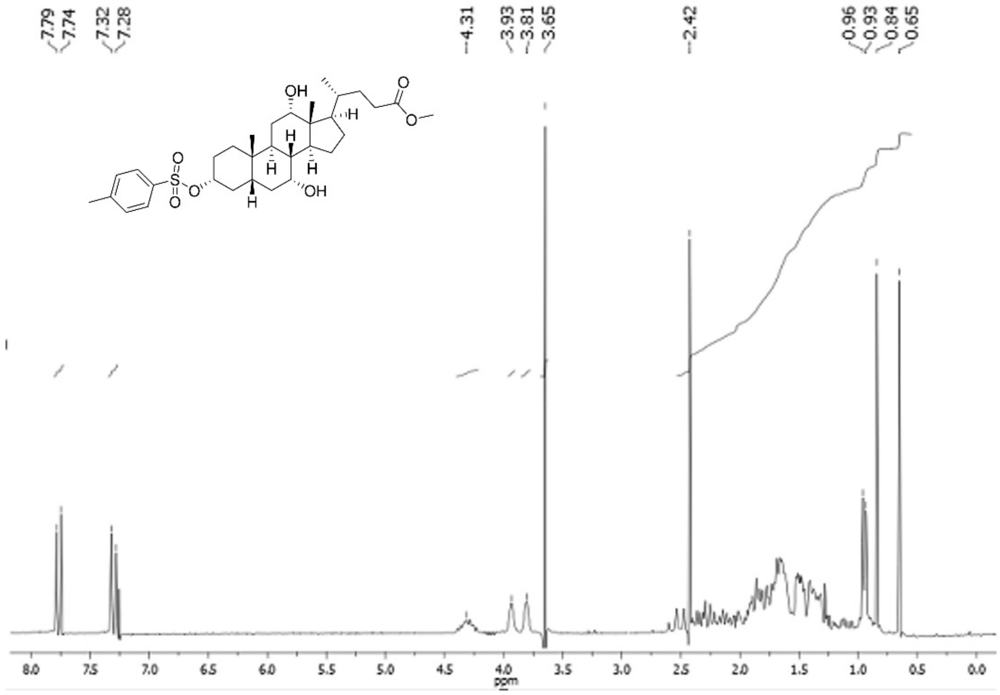

Figure S9. ^{1}H-NMR compound 9

^{1}H-NMR (200 MHz, $CDCl_3$) compound **9**.

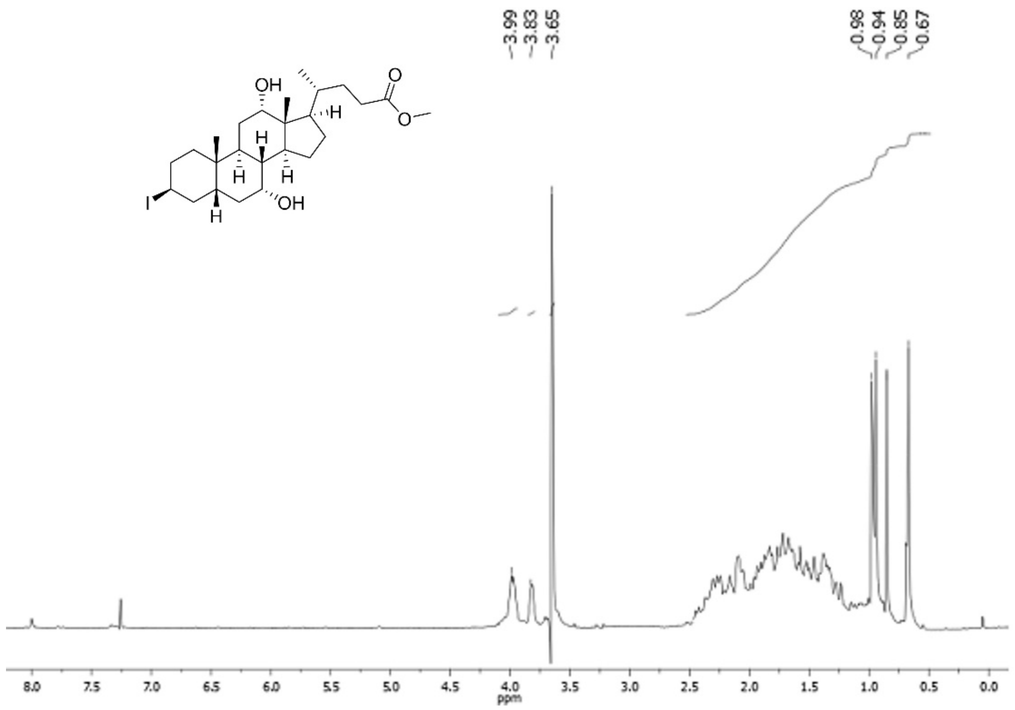

Figure S10. ^{1}H-NMR compound 10

^{1}H-NMR (200 MHz, $CDCl_3$) compound **10**.

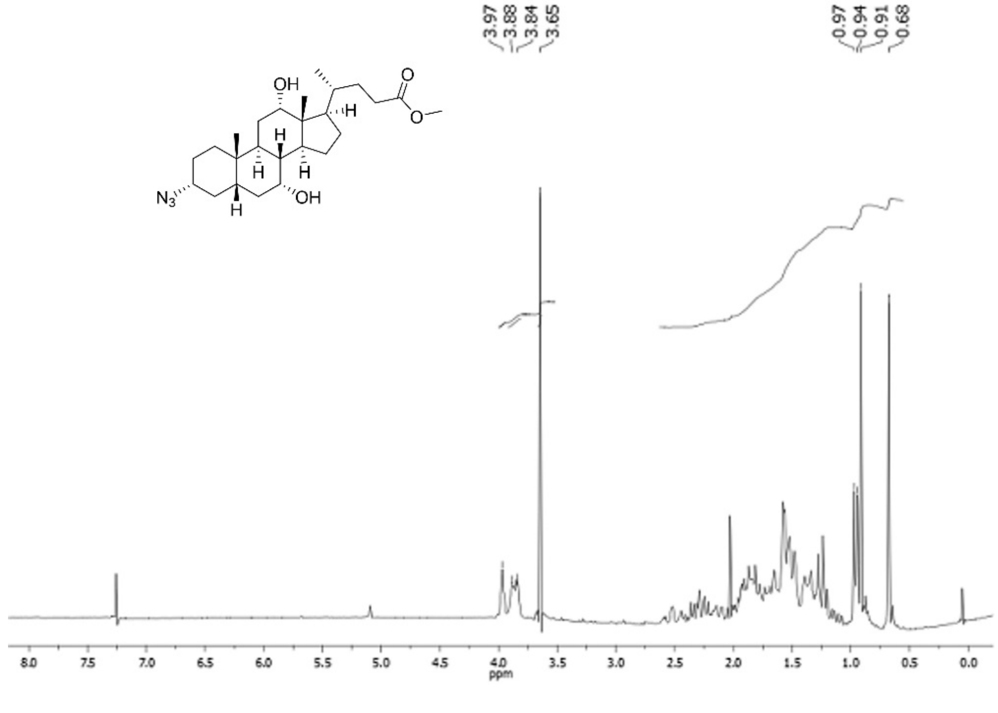

Figure S11. ^{1}H-NMR compound 11

^{1}H-NMR (200 MHz, CD_3OD) compound **11**.

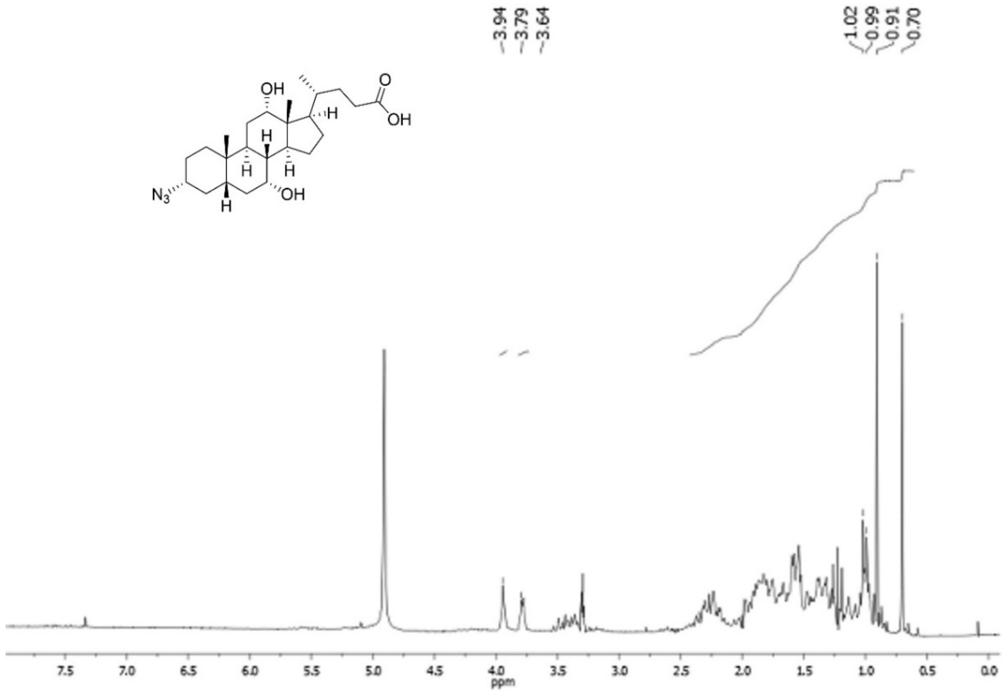

Figure S12. ^{1}H-NMR and HRMS spectra of compound 12

^{1}H-NMR (400 MHz, DMSO-d$_6$) compound **12 NIRBAD-2**

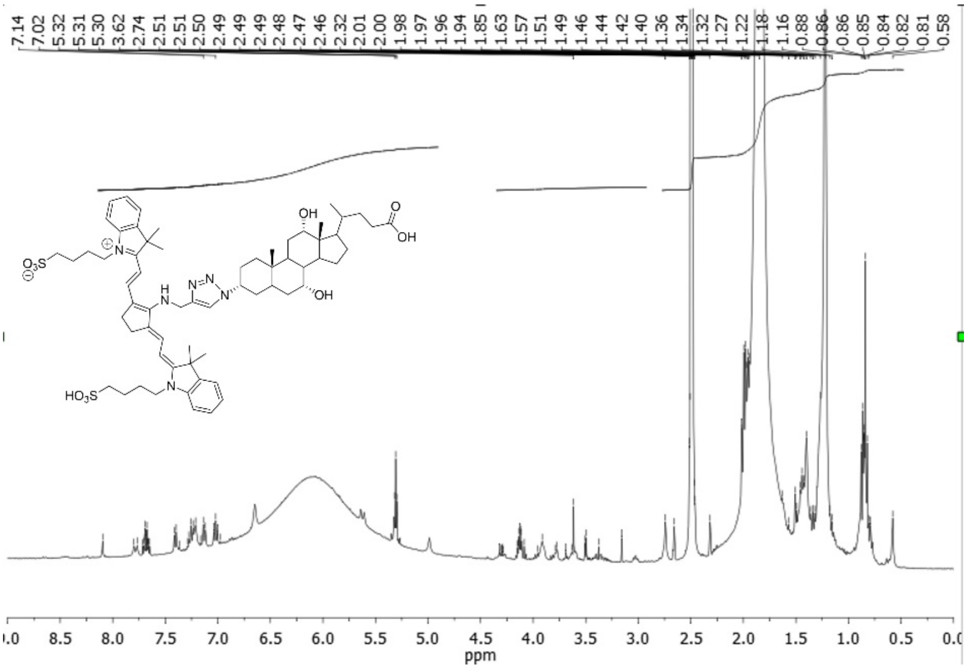

^{1}H-NMR (400 MHz, DMSO-d$_6$) compound **12 NIRBAD-2**

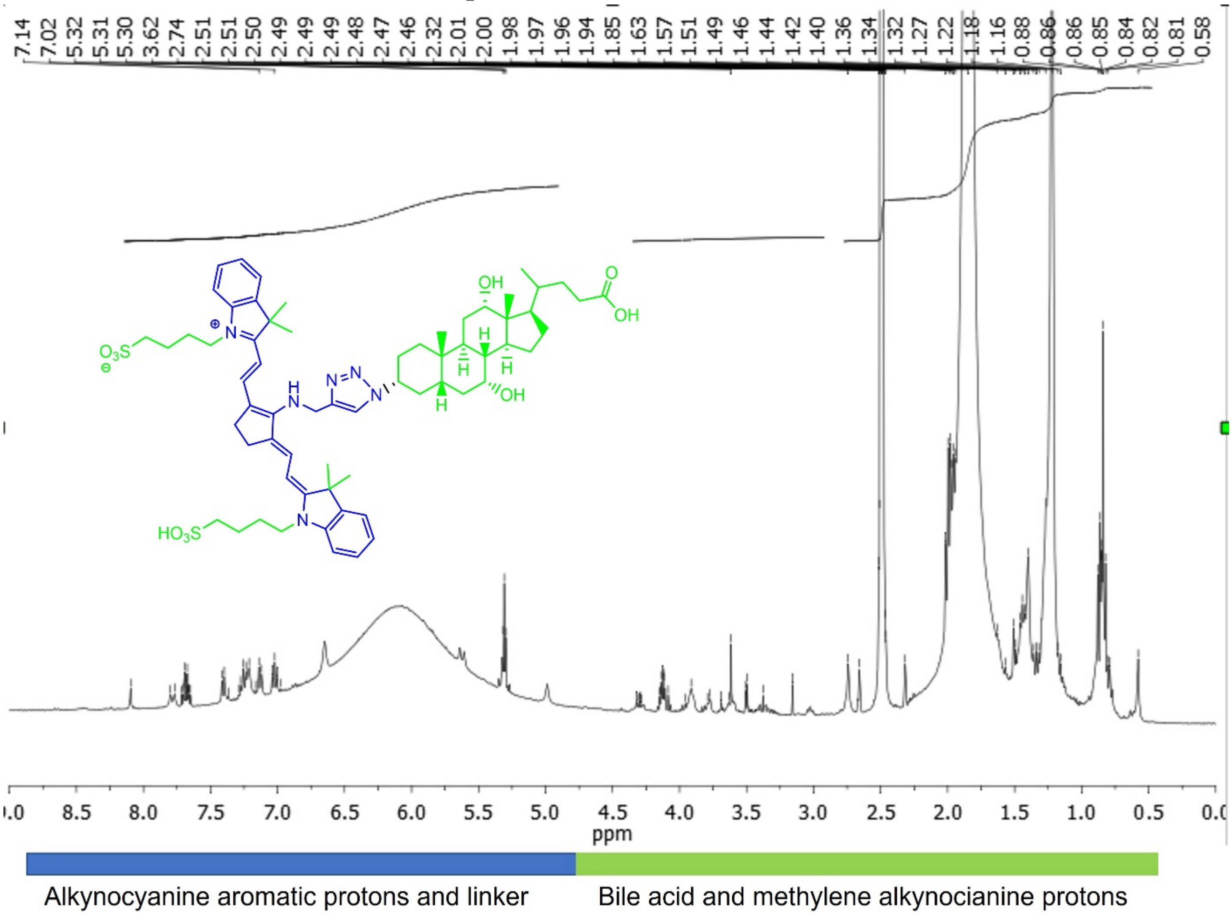

HRMS spectrum of compound **12 NIRBAD-2**

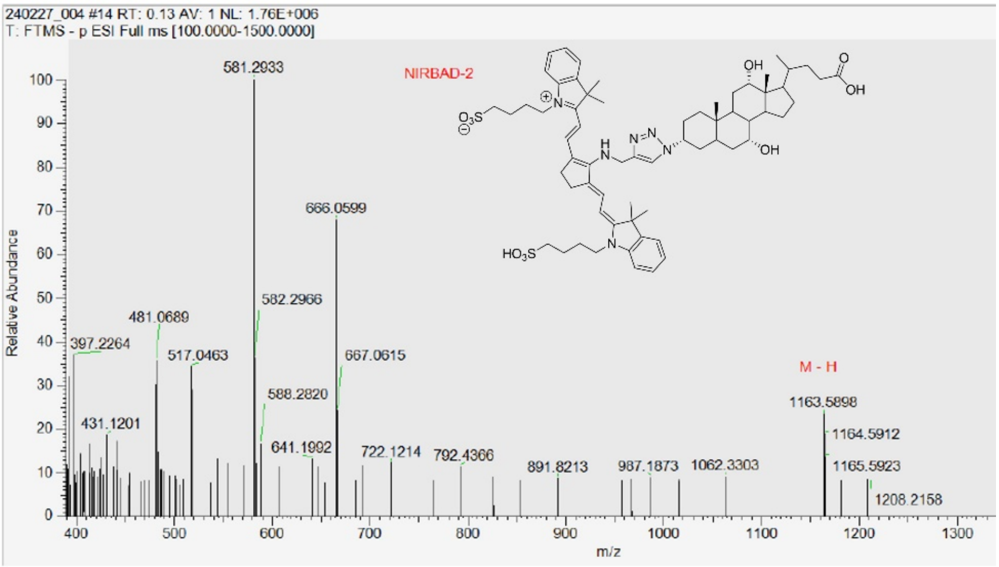

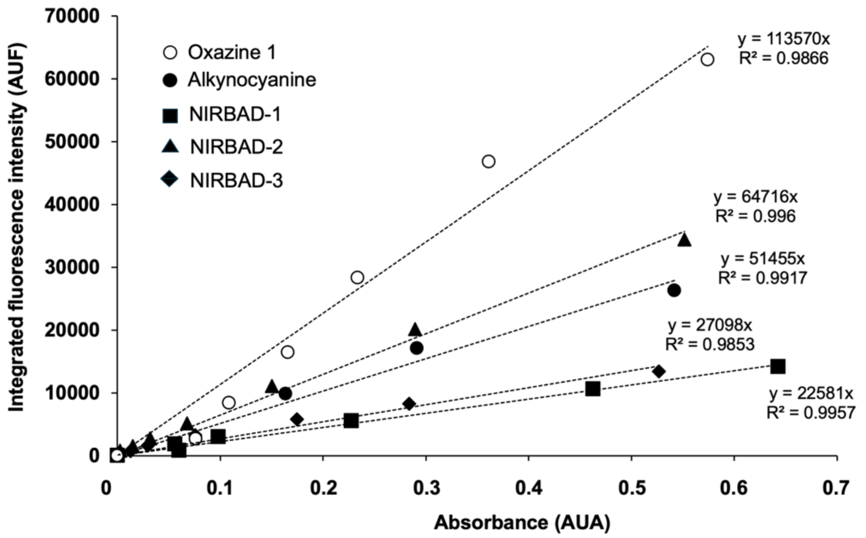

Figure S13. Relative quantum yield of NIRBAD-1, NIRBAD-2, NIRBAD-3, and alkynocyanine related to oxazine 1 as determined by the Parker-Rees' method. Compounds were dissolved in ethanol to obtain an initial stock solution without a solvent-related quenching effect, and serial dilutions were performed to acquire absorbance and fluorescence values. Values are means from measurements carried out in 3 separate preparations. AUF, arbitrary units of fluorescence; AUA, arbitrary units of absorbance.

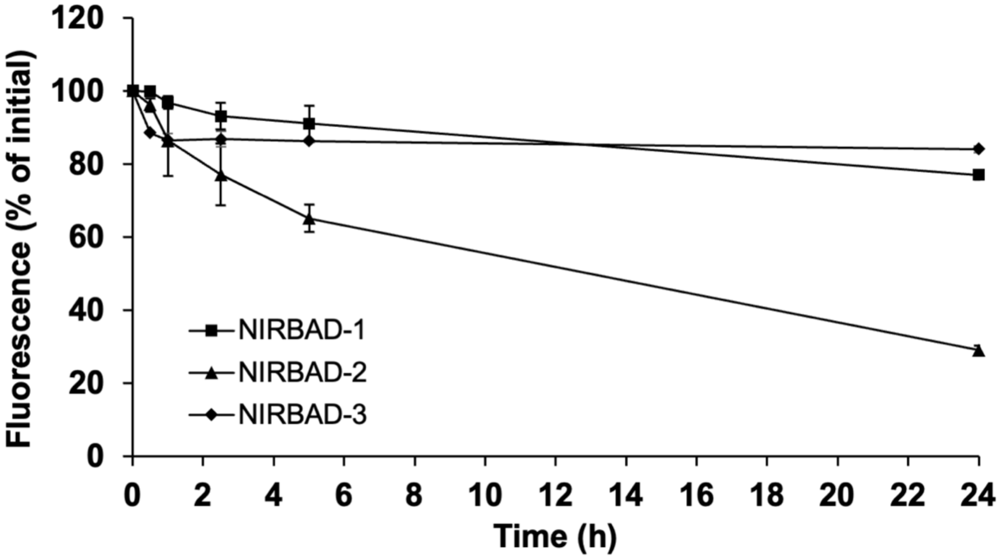

Figure S14. Photostability analysis of NIRBAD-1, NIRBAD-2, and NIRBAD-3 under conditions of uptake experiments at 37°C, in the dark. Compounds were dissolved in PBS buffer (pH 7.4) containing 1% DMSO to a 10 µM final concentration. Values are represented as means of sequential measurements in 3 separate preparations.

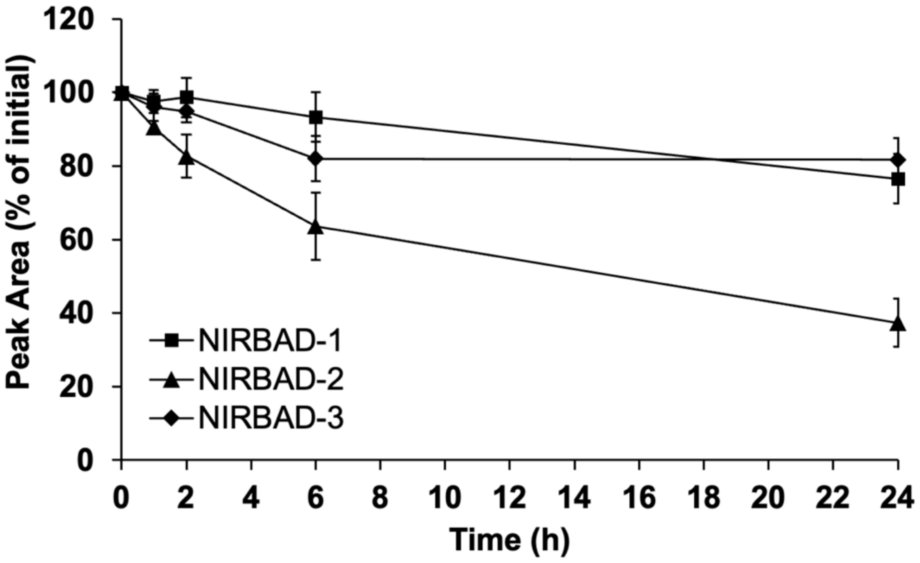

Figure S15. Chemical stability of NIRBAD-1, NIRBAD-2, and NIRBAD-3, which were dissolved in uptake medium (96 mM NaCl, 0.8 mM $MgSO_4$, 5.3 mM KCl, 1.1 mM KH_2PO_4, 1.8 mM $CaCl_2$, 11 mM D-glucose, 10 mM HEPES/Tris, pH 7.4) used in transport experiments. Their stability at 37°C maintained in the dark for up to 24 h, was determined by HPLC-MS/MS by monitoring the area of the peak corresponding to each specific molecular ion (606 *m/z*, 583 *m/z*, and 605 *m/z* for NIRBAD-1, -2 and -3, respectively). Values are represented as means of sequential measurements in 3 separate preparations.
